# International Environmental Impact of CKD Care

**DOI:** 10.1016/j.ekir.2025.10.019

**Published:** 2025-10-30

**Authors:** Katherine A. Barraclough, Aleix Cases, Matthew J. Eckelman, Celine Germond-Duret, Carmine Zoccali, Nina Embleton, Antony Wright, Luke Hubbert, Lindsay Nicholson, Salvatore Barone, Claudia Cabrera, Juan Jose Garcia Sanchez, Viknesh Selvarajah, Roberto Pecoits-Filho

**Affiliations:** 1 Department of Nephrology, Royal Melbourne Hospital, Parkville, Victoria, Australia; 2 Department of Medicine, University of Melbourne, Parkville, Victoria, Australia; 3 Nephrology Unit, Hospital Clínic de Barcelona, Barcelona, Spain; 4 Department of Civil and Environmental Engineering, Northeastern University, Boston, Massachusetts, USA; 5 School of Global Affairs, Lancaster University, Lancaster, UK; 6 Renal Research Institute, New York, New York, USA; 7 Institute of Biology and Molecular Genetics, Ariano Irpino, Italy; 8 Associazione Ipertensione Nefrologia Trapianto Renale, c/o Nefrologia, Grande Ospedale Metropolitano, Reggio Calabria, Italy; 9 Maverex Limited, Newcastle upon Tyne, UK; 10 Global Medical Affairs, AstraZeneca, Boston, Massachusetts, USA; 11 Emerging Medicines, BioPharmaceutical Medical AstraZeneca, Gothenburg, Sweden; 12 Global Market Access and Pricing, AstraZeneca, Barcelona, Spain; 13 Research and Early Development, Cardiovascular, Renal and Metabolism, BioPharmaceuticals R&D, AstraZeneca, Cambridge, UK; 14 Clin Epi Program Area, Arbor Research Collaborative for Health, Ann Arbor, Michigan, USA; 15 Department of Medicine, Pontificia Universidade Catolica do Parana, Curitiba, Brazil

**Keywords:** chronic kidney disease, environmental impact, greenhouse gas emissions, kidney replacement therapy, life cycle assessment

## Abstract

**Introduction:**

Data reporting the environmental impact of the overall chronic kidney disease (CKD) care pathway are limited.

**Methods:**

We performed a life cycle assessment (LCA) of CKD stages 1 to 5, with a primary focus on greenhouse gas (GHG) emissions and a secondary aim of quantifying broader environmental effects. The main scope estimated annual environmental impacts in the USA and UK, both per patient and for the total CKD population, with 8 additional countries included in exploratory analyses. Model inputs (annual health care resource use; travel distance; energy mix; and heating, cooling, and lighting requirements) were country-specific, where available. Environmental impacts by stage were calculated using the ReCiPe impact assessment method.

**Results:**

In the USA and UK, annual per-patient GHG emissions increased with CKD stage, from 1.9 to 7.8 tonnes and 0.4 to 5.1 tonnes of carbon dioxide equivalents (CO_2_e), respectively, with similar trends for other environmental impacts. Total annual GHG emissions were 30.6 and 1.8 megatonnes CO_2_e in the USA and UK, respectively, with stage 3 contributing the greatest proportion. Hospitalization drove emissions for stages 1 to 4, for stage 5 on supportive care, and for the prevalent transplant population. For patients receiving kidney replacement therapy (KRT), choice of modality drove GHG emissions. Although only 6.7% of the US CKD population and 2.6% of the UK population received KRT, this accounted for 15.2% and 11.1% of national CKD emissions, respectively, largely from thrice-weekly in-center hemodialysis (HD).

**Conclusion:**

This research provides insights into the overall environmental burden of CKD and impact hotspots, enabling the development of targeted interventions that reduce emissions.

## Graphical abstract

**Figure:**
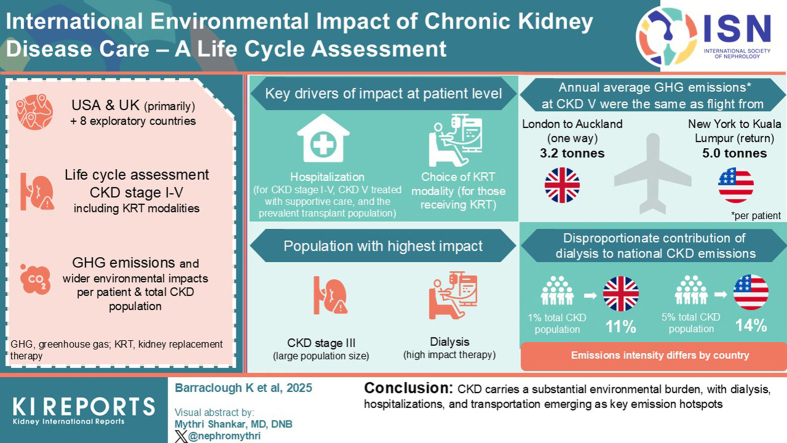

Climate change is having an increasingly devastating impact on the natural environment and human health and wellbeing.^1^ Although energy, transport, agriculture, and manufacturing are well-recognized contributors to climate change,^2^^,^^3^ the health care sector also plays a significant role, accounting for 4.4% to 5.2% of global GHG emissions.^1^^,^^4^, ^5^, ^6^, ^7^, ^8^ In response, there is a growing interest among governments, policy makers, and clinicians in building environmentally sustainable health care systems.^9^

CKD is recognized as a leading public health problem, affecting > 850 million people worldwide.^10^ By 2040, it is projected that CKD will be the fifth highest cause of years of life lost globally,^11^ driven by aging populations, increasing numbers with contributory diseases such as diabetes and hypertension,^12^ and increasing access to KRTs.^12^ As kidney function declines, the health and economic burden of CKD intensifies, with patients with kidney failure experiencing the worst clinical outcomes, the lowest quality of life, and the highest health care costs.^13^ However, there is a substantial health care resource burden at all CKD stages, including frequent health care contact, blood tests, and procedures. Although data exists on the environmental impact of specific aspects of kidney care, such as the carbon footprint of HD^14^, ^15^, ^16^, ^17^, ^18^, ^19^ and peritoneal dialysis (PD),^18^^,^^20^ there is a paucity of data on the environmental impact of the broader CKD care pathway, including comparative data on the different CKD stages and KRT modalities.

LCA is a systematic method for evaluating the environmental impact of a product, process, or service throughout its entire life cycle. Although LCA is commonly associated with industries such as manufacturing and agriculture, its application in health care is gaining attention because of increasing awareness of the environmental impact of health care activities and commitments to decarbonize health systems. Traditionally, health care LCAs have focused on GHG emissions, reported as CO_2_e. However, besides GHG emissions, health care activities, including kidney care, have other environmental impacts that LCAs can account for. These include impacts such as water depletion, land use, ozone depletion and fine particulate matter pollution, using impact assessment methods such as CML,^21^ ReCiPe,^22^ and TRACI methods (Supplementary Table S1).^23^ In the context of CKD, these impacts arise not only from dialysis but also from the broader care pathway, including routine laboratory testing, specialist visits, long-term medication use, and associated supply chains, all of which contribute to environmental harm beyond CO_2_e. To date, no LCA has estimated GHG emissions associated with the entire CKD care pathway or considered other environmental impacts.

We previously developed a framework to estimate the environmental impact associated with individual aspects of health care delivery across 10 different geographies, using CKD as an example.^24^ Building on this, the current analysis provides a system-level assessment of environmental impacts across the CKD care continuum, using real-world data for health care resource use and epidemiological data. The primary aim of this study was to identify GHG emissions and “hotspots” across all CKD stages and KRT modalities, and to compare countries to highlight best practices in delivering low-carbon CKD care. A secondary aim of the analysis was to quantify wider environmental impacts.

## Methods

### Study Overview

**Figure 1:**
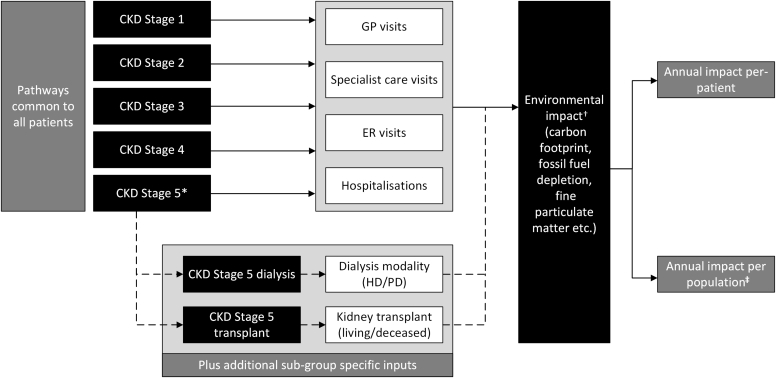
Study overview. ∗CKD stage 5 comprises the following 3 patient subgroups: supportive care, defined as management of kidney failure without dialysis or transplantation; dialysis, in which patients can undergo HD (in-center/satellite or at-home) or PD (automated PD or continuous ambulatory PD), with each of the different dialysis modalities modelled; and transplant*:* further divided between peritransplant, representing a patient who has undergone transplantation within that year; or prevalent transplant, where a patient has received a transplant and now receives routine maintenance care. †The environmental impact is reported according to the categories from ReCiPe guidelines, to translate emissions and resource extractions into environmental impact scores. ‡Scaled up to the Inside CKD population data. CKD, chronic kidney disease; ER, emergency room; GP, general practitioner; HD, hemodialysis; PD, peritoneal dialysis.

An LCA was conducted to estimate the environmental burden at all CKD stages aligned to ISO 14040/14044 international standards, which provide a globally accepted framework for conducting and reporting LCAs in a consistent and transparent manner. Modules were built representing different resource inputs that comprise the CKD treatment pathway, including annual health care visits, hospitalization events, and KRT (stratified by modality) (Figure 1), as previously described.^24^

Evaluations were carried out at the patient- and population-level across 10 countries selected for their data availability and inclusion in Inside CKD, a modelling framework designed to simulate the projected global prevalence and burden of CKD. Inside CKD was chosen for its detailed, stage-specific prevalence estimates and consistent stratification by CKD stage and treatment modality, enabling robust cross- comparisons. The selected countries were Australia, Belgium, Brazil, Germany, Japan, Italy, the Netherlands, Spain, the UK, and the USA. Results were stratified by CKD stage and KRT and reported as annual impacts.

For the population-level analyses, patient-level data were scaled up according to country-specific prevalence data for the diagnosed CKD population, reported by Inside CKD.^25^ The model structure and calibration process have been previously described in detail.^24^ Because Inside CKD does not stratify stage 5 by treatment type, we supplemented its figures with modality-specific counts from national registries, where available. Because of limited data availability, the UK and USA formed the main analyses, with the remaining countries forming an exploratory analysis.

### Patient Population

The patient population comprised adults diagnosed with CKD stage 1 to 5, as defined by the Kidney Disease Improving Global Outcomes classification system.^26^ CKD stage 5 was categorized into supportive care (defined as management of kidney failure without dialysis or transplantation), HD, PD, and transplantation. Both at-home and in-center HD were considered, as were automated PD (APD) and continuous ambulatory PD (CAPD). The specific KRT regimens modelled are shown in Table 1. Transplantation was further divided into the peritransplant period (patients undergoing transplant surgery, and a 1-year follow-up) and the posttransplant maintenance period (“prevalent transplant,” patients with a functioning kidney transplant). Based on UK Renal Registry data, it was assumed that 1.5% of patients across all countries would receive supportive care.^27^ Dialysis modality percentages were derived from country-specific registry data for Australia, Brazil, Germany, Japan, the Netherlands, Spain, the UK, and the USA. For Belgium and Italy, German data were used because of lack of country-specific data. In the absence of data on at-home HD rates in Germany, we assumed it to be 20% of the PD population, based on published data reporting home HD to be 1% compared with 4% of 5% of PD.^28^ Proportions of patients receiving APD versus CAPD were not reported for Japan and were assumed to be evenly distributed. KRT modality proportions at CKD stage 5 are outlined in Supplementary Table S2; and the prevalence data are presented in Supplementary Table S3.

**Table 1:** Dialysis regimens assessed within this study

Modality	Treatment frequency, volume	Duration of heating, cooling, and lighting applied^a^
At-home hemodialysis	4 x weekly	8 h per treatment
In-center hemodialysis	3 x weekly	5 h per treatment
Continuous ambulatory peritoneal dialysis	10 L	4 h daily
Automated peritoneal dialysis	12.5 L	7.5 h daily

a Duration includes both set-up, wait time, treatment time, and machine shut down.

### Model Structure and Life Cycle Inventory Analysis

LCA Software for Experts (formerly GaBi software)^29^ was used to model the pathway using inputs from ecoinvent Life Cycle Inventory database version 3.8.^30^ Where exact matches for the materials, energy, and emissions related to any aspect of the CKD treatment pathway were unavailable in ecoinvent’s database, a suitable proxy was chosen that best matched the product characteristics. Details of the study boundary have been published elsewhere.^24^

Model inputs for annual health care resource use; travel distance; energy mix; and heating, cooling, and lighting requirements were individualized for each country, where data were available. Detailed methods for developing the modules are reported in the Supplementary Methods. Briefly, it was assumed that modes of transport (car, bus, train, ambulance, or walking) would change based on CKD stage and destination. Country-specific energy mixes were derived from ecoinvent database.^30^ These values were then scaled up according to the total energy used for heating, cooling, and lighting for health care areas, which was calculated based on kWh/m^2^ and areas/patient for each type of health care site, obtained from various sources.^31^, ^32^, ^33^, ^34^, ^35^, ^36^, ^37^, ^38^ Once modules had been built up using inputs from the ecoinvent database, as reported previously,^24^ each life cycle stage was then scaled up according to annual health care resource data, such as numbers of general practitioner visits, or hospitalizations, as identified by literature review.

### Life Cycle Impact Assessment

The environmental impact for each CKD stage was calculated using the ReCiPe 2016 v1.1 impact assessment methodology, selected because of its wide global applicability.^22^ This method converts lifecycle inventory data, such as health care resource use, patient travel, and consumables into various environmental impact categories, including GHG emissions, fossil depletion, and fine particulate matter formation. These midpoint indicators, which represent intermediate environmental effects often linked to specific environmental problems such as climate change, were analyzed using the hierarchist time perspective of 100 years. This perspective emphasizes the long-term consequences of environmental impacts, focusing on the cumulative effects of emissions and resource depletion over extended periods, with the aim of ensuring that future generations are not unduly burdened by current environmental decisions.

### Main analysis and Exploratory Analysis

The main analyses assessed the annual environmental impact of CKD across all stages in the USA and the UK, primarily based on data from the DISCOVER CKD program, which reports real-world evidence of health care resource use, stratified by CKD stage (Supplementary Tables S4 and S5).^39^^,^^40^ DISCOVER CKD captures data for diagnosed patients identified not only through hospitalizations and complications but also through outpatient testing, prescription data, and primary care records, helping to account for those at the earlier stage of disease. Additional exploratory analyses were carried out for the remaining 8 countries. For these countries, country-specific data was used for heating, cooling, lighting, and travel distance to health care sites, whereas health care resource use data from the UK and USA were applied because of limited country-specific data.

### Sensitivity Analysis

The robustness of the results was assessed using one-way sensitivity analyses, varying key parameters individually to examine their impact on overall environmental outcomes. Parameters tested included assumptions about telemedicine and electric transport use; distances travelled; heating, cooling, and lighting values to reflect different climates, the number and length of hospitalization events, and inclusion of medications. This approach highlighted the variables with the greatest influence on results.

### Model Validation

Data sources, assumptions, results, and interpretation were based on the best available peer-reviewed papers and validated with expert opinions.

## Results

### Main Analysis

#### Patient-Level Emissions

**Figure 2:**
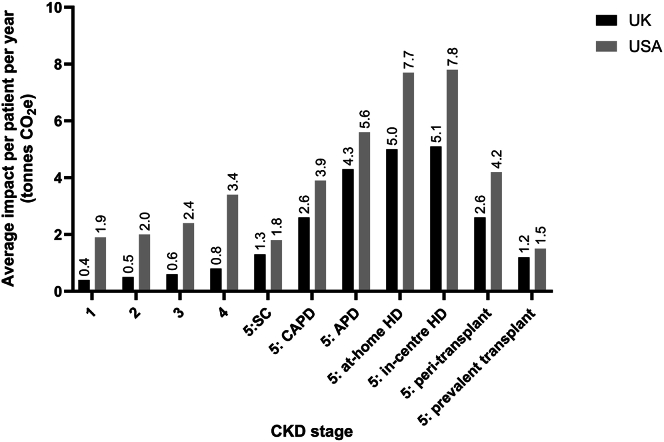
As CKD progresses, greenhouse gas emissions increase per patient per year, in the UK and USA. APD, automated peritoneal dialysis; CAPD, continuous ambulatory peritoneal dialysis; CKD, chronic kidney disease; HD, hemodialysis; SC, supportive care; tonnes CO_2_e, metric tonnes carbon dioxide equivalents.

At the patient level, annual GHG emissions increased with progression of CKD in both the USA and the UK. In the USA, emissions ranged from 1.9 tonnes CO_2_e/patient/yr at CKD stage 1 to 7.8 tonnes CO_2_e for thrice-weekly in-center HD (Figure 2; Supplementary Table S6). In the UK, annual GHG emissions ranged from 0.4 tonnes CO_2_e/patient/yr at CKD stage 1 to 5.1 tonnes CO_2_e for thrice-weekly in-center HD (Figure 2; Supplementary Table S7).

**Figure 3:**
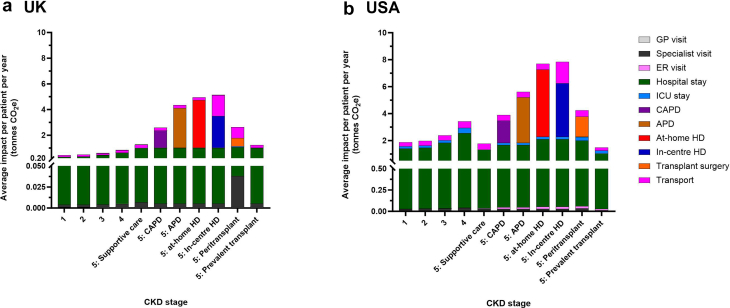
At the patient-level, health care drivers of annual greenhouse gas emissions were hospitalizations and kidney replacement therapy modality in (a) the UK and (b) USA. APD, automated peritoneal dialysis; CAPD, continuous ambulatory peritoneal dialysis; CKD, chronic kidney disease; ER, emergency room; HD, hemodialysis; ICU, intensive care unit; SC, supportive care; tonnes CO_2_e, metric tonnes carbon dioxide equivalents.

Hospitalizations were the primary driver of GHG emissions for CKD stages 1 to 4, for CKD stage 5 treated with supportive care, and for the prevalent transplant population (Figure 3). These accounted for 67.9% to 75.5% of total emissions in the USA, and 66.6% to 80.2% in the UK.

At CKD stage 5, where patients were receiving KRT, the choice of modality was the key driver of GHG emissions. HD, whether at-home or in-center, resulted in the highest GHG emissions, followed by APD then CAPD. Transplantation had the lowest impact among all the KRT modalities, with GHG emissions being higher in the peritransplant period than in the latter “prevalent” transplant phase (Figure 3). In the latter group, GHG emissions were comparable with those observed in earlier stages of CKD in both the USA and the UK.

#### Population-Level Emissions

**Figure 4:**
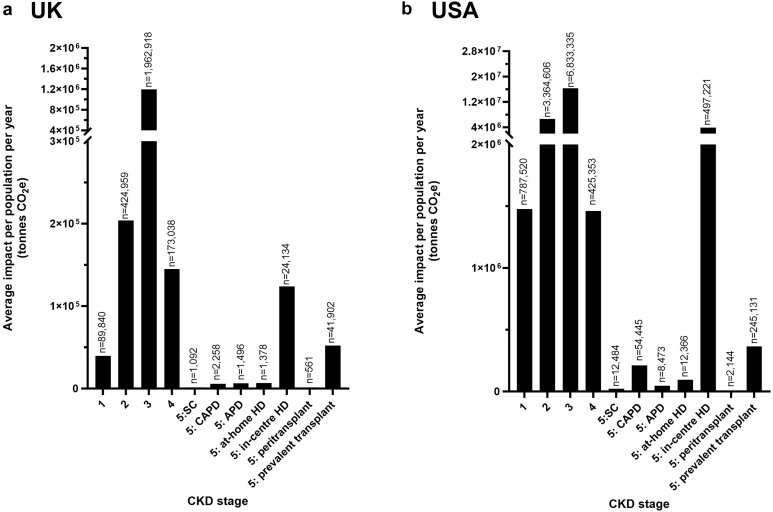
At the population level, CKD stage 3 has the highest greenhouse gas emissions in (a) the UK and (b) USA annually. APD, automated peritoneal dialysis; CAPD, continuous ambulatory peritoneal dialysis; CKD, chronic kidney disease; HD, hemodialysis; *n*, denotes the number of patients at each stage; SC, supportive care; tonnes CO_2_e, metric tonnes carbon dioxide equivalents.

**Figure 5:**
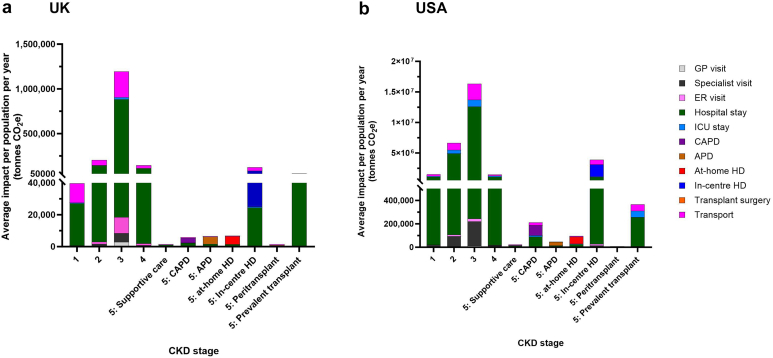
At the population level, annual greenhouse gas emissions are driven by CKD stage 3 in (a) the UK and (b) USA. APD, automated peritoneal dialysis; CAPD, continuous ambulatory peritoneal dialysis; CKD, chronic kidney disease; ER, emergency room; GP, general practitioner; HD, hemodialysis; ICU, intensive care unit; SC, supportive care; tonnes CO_2_e, metric tonnes carbon dioxide equivalents.

In total, individuals with diagnosed CKD were responsible for 30.5 megatonnes CO_2_e annually in the USA and 1.8 megatonnes CO_2_e in the UK. At the population-level, CKD stage 3 was the largest contributor to GHG emissions, accounting for 53% (16.3 megatonnes CO_2_e) of total CKD emissions in the USA and 67% (1.2 megatonnes CO_2_e) in the UK. This was because of the high numbers of individuals diagnosed with CKD stage 3 relative to other CKD stages (56% and 72% of the total USA and UK populations, respectively) (Figures 4 and 5 and Table 2).

**Table 2:** Percentage contributions of total GHG emissions based on the prevalent CKD population in the UK and USA from Inside CKD data

UK					USA			
Stage	Prevalence	% total prevalence	CO_2_e (tonnes)	% CO_2_e contribution	Prevalence	% total prevalence	CO_2_e (tonnes)	% CO_2_e contribution
1.0	89,840	3.3%	39,700	2.2%	787,520	6.4%	1,480,000	4.9%
2.0	424,960	15.6%	204,000	11.5%	3,364,606	27.5%	6,620,000	21.7%
3.0	1,962,918	72.1%	1,190,000	67.0%	6,833,335	55.8%	16,300,000	53.4%
4.0	173,038	6.4%	145,000	8.2%	425,353	3.5%	1,460,000	4.8%
5:SC	1092	0.0%	1390	0.1%	12,484	0.1%	21,900	0.1%
5: PD (CAPD)	2258	0.1%	5820	0.3%	54,445	0.4%	211,000	0.7%
5: PD (APD)	1496	0.1%	6500	0.4%	8473	0.1%	47,500	0.2%
5: at-home HD	1378	0.1%	6830	0.4%	12,366	0.1%	95,100	0.3%
5: in-center HD	24,134	0.9%	124,000	7.0%	497,221	4.1%	3,900,000	12.8%
5: peritransplant	561	0.0%	1480	0.1%	2144	0.0%	9080	0.0%
5: prevalent transplant	41,902	1.5%	52,000	2.9%	245,131	2.0%	366,000	1.2%
Total population	2,723,576	Total CO_2_e (tonnes)	1,776,720	100%	12,243,079	Total CO_2_e (tonnes)	30,510,580	100%

APD, automated peritoneal dialysis; CAPD, continuous ambulatory peritoneal dialysis; CKD, chronic kidney disease; CO_2_e, carbon dioxide equivalents; GHG, greenhouse gas; HD, hemodialysis; PD, peritoneal dialysis; SC, supportive care.

Despite those receiving dialysis making up just 4.7% of the prevalent CKD population in the USA and 1.1% in the UK (Table 2), these groups imposed a high GHG emissions impact, accounting for 14% of total CKD emissions in the USA (4.3 megatonnes CO_2_e) and 8% in the UK (0.1 megatonnes). Thrice-weekly in-center HD was the main contributor, responsible for 92% of dialysis emissions (3.9 megatonnes CO_2_e) and 13% of total CKD emissions in the USA; and 87% of dialysis emissions (0.1 megatonnes CO_2_e) and 7% of total CKD emissions in the UK (Table 2).

**Figure 6:**
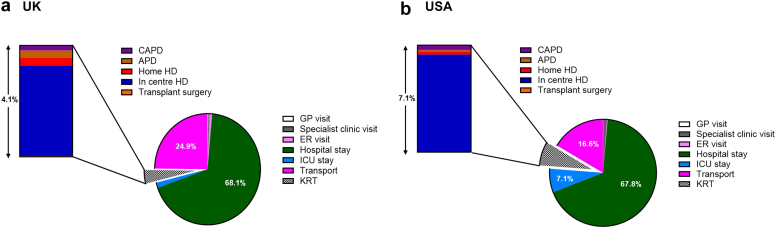
Hospitalization and transport drive the greenhouse gas emissions across the total chronic kidney disease population in (a) the UK and (b) USA. APD, automated peritoneal dialysis; CAPD, continuous ambulatory peritoneal dialysis; ER, emergency room; GP, general practitioner; HD, hemodialysis; ICU, intensive care unit; KRT, kidney replacement treatment; tonnes CO_2_e, metric tonnes carbon dioxide equivalents.

Similar to patient-level analyses, hospitalizations had the highest impact at the population-level for CKD 1 to 4, CKD stage 5 with supportive care, and prevalent transplant (67.9%–75.5% in the USA, 66.6%–80.2% in the UK). In those receiving KRT, treatment modality drove GHG emissions, with in-center HD having the largest footprint (Figure 5). Across the total CKD population, hospitalizations, and transport accounted for 85% of the total GHG emissions in the USA (68% and 17%, respectively) and 93% in the UK (68% and 25%, respectively) (Figure 6; Supplementary Table S8).

#### Other Environmental Impact Categories

As with GHG emissions, other environmental impacts increased with increasing CKD stage, both in the USA (Supplementary Table S9) and UK (Supplementary Table S10). At the population-level, CKD stage 3 had the greatest environmental impact across all categories (Supplementary Tables S11 and S12).

### Exploratory Analysis: Extending the Scope Beyond the USA and UK

At the patient-level, GHG emissions increased with CKD stage for all assessed countries (Supplementary Figure S1). At-home and in-center HD were the largest contributors to GHG emissions across the whole CKD pathway.

When using the UK dataset for health care resource use and scaled to the prevalent population for each country, total GHG emissions ranged from 0.3 megatonnes CO_2_e in Belgium to 10.6 megatonnes CO_2_e in Japan. Using the USA dataset, total GHG emissions ranged from 0.9 megatonnes CO_2_e in the Netherlands) to 21.9 megatonnes CO_2_e in Japan. At the population-level, GHG emissions were greatest at CKD stage 3 (Supplementary Table S13).

### Sensitivity Analyses

Incorporating telemedicine into the care pathway for 50% of general practitioner and specialist care visits led to a 2% to 11% reduction in per patient GHG emissions in the USA and a 1% to 15% reduction in the UK, compared with baseline emissions, depending on CKD stage. In Tables 3 and 4, we present the details of the reductions in GHG emissions observed from switching a proportion of petrol vehicles to electric vehicles, optimizing heating, cooling, and lighting requirements, and reducing health care stays and health visits. When including the impact of medications in analyses, GHG emissions increased by up to 141% in the USA and 172% in the UK. However, these increases should be interpreted with caution, because modelling was based on limited available data. The results of the sensitivity analyses per population are shown in Supplementary Tables S14, S15.

**Table 3:** Sensitivity analysis shows how altering the different model parameters in the USA affects annual GHG emissions per patient

Model parameter	GHG emissions (tonnes CO_2_e) (% difference vs. base case)										
	1	2	3	4	5: SC	5: CAPD	5: APD	5: at-home HD	5: in-center HD	5: peritransplant	5: prevalent transplant
Base case	1.9	2.0	2.4	3.4	1.8	3.9	5.6	7.7	7.8	4.2	1.5
50% telemedicine	1.8 (93.3%)	1.8 (93.1%)	2.2 (93.4%)	3.2 (94.0%)	1.6 (89.9%)	3.7 (95.8%)	5.4 (97.0%)	7.5 (97.9%)	7.7 (97.9%)	4.1 (95.6%)	1.4 (95.1%)
50% electric vehicles	1.8 (97.1%)	1.9 (97.1%)	2.3 (97.5%)	3.4 (97.5%)	1.7 (95.6%)	3.8 (98.1%)	5.5 (98.8%)	7.6 (99.1%)	7.6 (96.6%)	4.2 (97.9%)	1.5 (97.1%)
50% heating/cooling/lighting	1.3 (66.7%)	1.3 (66.6%)	1.6 (66.1%)	2.3 (65.7%)	1.2 (69.4%)	2.9 (74.9%)	4.4 (79.0%)	6.5 (84.6%)	6.2 (78.5%)	3.1 (72.2%)	1.0 (66.3%)
150% heating/cooling/lighting	2.5 (133.3%)	2.6 (133.3%)	3.2 (134.0%)	4.6 (134.1%)	2.3 (130.8%)	4.9 (125.2%)	6.8 (121.0%)	8.9 (115.5%)	9.5 (121.5%)	5.4 (127.9%)	2.0 (134.0%)
50% distance	1.7 (92.8%)	1.8 (92.6%)	2.2 (92.9%)	3.2 (93.7%)	1.6 (89.9%)	3.7 (95.5%)	5.4 (96.8%)	7.5 (97.6%)	7.1 (90.4%)	3.6 (85.9%)	1.4 (94.5%)
150% distance	2.0 (107.2%)	2.1 (107.3%)	2.6 (107.2%)	3.7 (106.5%)	1.9 (110.4%)	4.1 (104.6%)	5.8 (103.2%)	7.9 (102.3%)	8.6 (109.6%)	4.8 (114.0%)	1.6 (105.8%)
50% hospital stay	1.1 (59.2%)	1.2 (59.5%)	1.4 (58.6%)	2.0 (57.9%)	1.1 (63.1%)	3.0 (76.7%)	4.7 (84.0%)	6.6 (85.4%)	6.7 (85.6%)	3.1 (73.6%)	0.9 (58.1%)
150% hospital stay	2.6 (140.8%)	2.8 (140.9%)	3.4 (141.5%)	4.9 (142.2%)	2.4 (137.1%)	4.8 (123.4%)	6.5 (116.1%)	8.8 (114.7%)	9.0 (114.4%)	5.4 (126.3%)	2.1 (142.0%)
50% health visits	1.1 (58.7%)	1.2 (59.0%)	1.4 (58.2%)	2.0 (57.3%)	1.1 (62.6%)	3.0 (76.5%)	4.7 (83.8%)	6.6 (85.2%)	6.7 (85.5%)	3.1 (73.4%)	0.9 (57.5%)
150% health visits	2.7 (141.3%)	2.8 (140.9%)	3.4 (141.9%)	4.9 (142.5%)	2.4 (137.7%)	4.8 (123.6%)	6.5 (116.2%)	8.8 (114.8%)	9.9 (126.0%)	5.4 (126.5%)	2.1 (142.7%)
Including medicine	2.2 (116.8%)	2.28 (116.0%)	2.8 (115.1%)	3.9 (112.6%)	2.3 (132.0%)	4.8 (122.6%)	6.5 (115.5%)	8.4 (108.7%)	8.5 (108.5%)	4.9 (114.5%)	2.1 (141.3%)

APD, automated peritoneal dialysis; CAPD, continuous ambulatory peritoneal dialysis; tonnes CO_2_e, metric tonnes carbon dioxide equivalents; GHG, greenhouse gas; HD, hemodialysis; SC, supportive care.

**Table 4:** Sensitivity analysis shows how altering the different model parameters in the UK affects annual GHG emissions per patient

Model parameter	GHG emissions (tonnes CO_2_e) (% difference vs. base case)										
	1	2	3	4	5: SC	5: CAPD	5: APD	5: at-home HD	5: in-center HD	5: peritransplant	5: prevalent transplant
Base case	0.4	0.5	0.6	0.8	1.3	2.6	4.3	5.0	5.1	2.6	1.2
50% telemedicine	0.4 (90.2%)	0.4 (90.9%)	0.6 (92.3%)	0.8 (92.4%)	1.2 (92.8%)	2.5 (97.3%)	4.3 (98.3%)	4.9 (98.7%)	5.1 (98.6%)	2.2 (85.1%)	1.2 (94.3%)
50% electric vehicles	0.4 (94.5%)	0.5 (95.1%)	0.6 (95.7%)	0.8 (95.8%)	1.2 (96.7%)	2.5 (98.5%)	4.3 (99.2%)	4.9 (99.3%)	4.9 (94.5%)	2.5 (94.2%)	1.2 (96.7%)
50% heating/cooling/lighting	0.3 (76.4%)	0.4 (75.7%)	0.5 (74.2%)	0.6 (73.8%)	0.9 (73.0%)	2.1 (82.2%)	3.8 (87.2%)	4.5 (90.4%)	4.4 (85.7%)	2.1 (80.5%)	0.9 (72.1%)
150% heating/cooling/lighting	0.5 (123.6%)	0.6 (124.5%)	0.8 (125.8%)	1.1 (126.4%)	1.6 (127.4%)	3 (117.8%)	4.9 (112.8%)	5.4 (109.6%)	5.9 (114.2%)	3.1 (119.3%)	1.6 (128.1%)
50% distance	0.4 (89.7%)	0.4 (90.5%)	0.6 (91.8%)	0.8 (91.9%)	1.2 (92.0%)	2.5 (96.9%)	4.3 (98.3%)	4.9 (98.5%)	4.3 (84.6%)	2.1 (80.1%)	1.2 (93.5%)
150% distance	0.5 (110.3%)	0.5 (109.4%)	0.7 (108.4%)	0.9 (108.1%)	1.4 (107.7%)	2.7 (103.1%)	4.4 (101.7%)	5 (101.5%)	5.9 (115.4%)	3.2 (119.6%)	1.3 (106.3%)
50% hospital stay	0.3 (65.8%)	0.3 (64.6%)	0.4 (62.3%)	0.5 (61.8%)	0.8 (60.6%)	2.1 (79.9%)	3.8 (88.2%)	4.4 (89.6%)	4.6 (90.0%)	2.1 (79.4%)	0.7 (59.1%)
150% hospital stay	0.6 (134.3%)	0.6 (135.3%)	0.8 (137.7%)	1.2 (138.3%)	1.8 (139.2%)	3.1 (119.8%)	4.9 (111.9%)	5.5 (110.4%)	5.7 (110.1%)	3.2 (120.4%)	1.8 (141.0%)
50% health visits	0.3 (65.3%)	0.3 (64.2%)	0.4 (61.8%)	0.5 (61.2%)	0.8 (59.9%)	2.1 (79.9%)	3.8 (87.9%)	4.4 (89.4%)	4.6 (89.8%)	2.1 (79.0%)	0.7 (58.4%)
150% health visits	0.6 (134.7%)	0.7 (135.9%)	0.8 (138.1%)	1.2 (138.3%)	1.8 (140.0%)	3.1 (120.2%)	4.9 (112.1%)	5.5 (110.6%)	5.7 (110.3%)	3.2 (120.8%)	1.8 (141.8%)
Including medicine	0.8 (171.8%)	0.8 (166.1%)	1.0 (159.5%)	1.3 (151.4%)	1.8 (143.9%)	3.4 (131.0%)	5.1 (118.3%)	5.6 (113.4%)	5.8 (113.0%)	3.3 (123.4%)	1.9 (149.0%)

APD, automated peritoneal dialysis; CAPD, continuous ambulatory peritoneal dialysis; GHG, greenhouse gas; HD, hemodialysis; SC, supportive care; tonnes CO_2_e, metric tonnes carbon dioxide equivalents.

## Discussion

Although the substantial clinical and socio-economic burden of CKD is well-recognized, there are limited data on its environmental impact. This study documents GHG emissions across the entire CKD care pathway, spanning stages 1 to 5. This provides a foundation for health care providers and policymakers to design interventions to reduce the environmental burden of CKD care. A summary of key findings, implications, and recommended actions are presented in Supplementary Table S16.

At the patient-level, GHG emissions were high across all CKD stages, increasing as the disease progresses. In the UK, CKD stage 1 added 12% to an average person’s annual footprint, increasing to 54% for stage 5 treated with in-center HD; in the USA, the corresponding figures were 9% and 99%.^41^ Hospitalizations determined GHG emissions for non-KRT patients, whereas for those patients undergoing KRT, modality choice was the key determinant; in-center HD incurred the highest emissions, followed by home HD, APD, CAPD, and then kidney transplantation. At the population-level, most emissions originated from CKD stage 3 because of its high prevalence. However, the burden of dialysis was substantial, despite the relatively low number of people receiving dialysis. Like the patient-level, hospitalizations were the primary source of emissions across all CKD stages. Patient travel contributed substantially because of frequent general practitioner and specialist visits.

Comparable findings have been reported by Nagai and colleagues, using an environmentally extended input-output model to assess GHG emissions across CKD stages in Japan. Their study similarly found that GHG emissions increased with CKD progression, with the highest per capita emissions observed among patients receiving dialysis, particularly in-center HD.^42^ These aligned results across different methodologies and health systems reinforce the validity and generalizability of the environmental burden posed by advanced CKD and underscore the urgent global need to decarbonize care pathways.

Taken together, these data highlight the critical importance of proactive screening and early management to slow CKD progression and reduce kidney failure. Key priorities include lifestyle changes (smoking cessation), strict glycemic and blood pressure control, and careful monitoring of patients with elevated albuminuria or proteinuria (urinary albumin-to-creatinine ratio or urinary protein-to-creatinine ratio, respectively), who are at higher risk of rapid progression to kidney failure.^43^^,^^44^ Routine use of renin-angiotensin system inhibitors remains foundational and expanding access to newer cardio-renoprotective therapies (sodium-glucose cotransporter-2 inhibitors, glucagon-like peptide-1 receptor agonists, and nonsteroidal mineralocorticoid receptor antagonists) is needed, particularly in regions with a rapidly growing CKD burden. Although these therapies carry direct GHG emissions from their manufacture, distribution, and disposal, their proven benefits in slowing CKD progression and reducing cardiovascular events are likely to yield substantial indirect environmental gains by reducing downstream health care resource use, including hospitalizations and the need for KRT, which, as our analysis shows, drive emissions in CKD care. For example, in the CREDENCE trial, the addition of a sodium-glucose cotransporter-2 inhibitor to routine therapy for patients with type 2 diabetes and CKD reduced per-participant annual GHG emissions from 196 kg CO_2_e to 157 kg CO_2_e by preventing hospital admissions and delaying dialysis.^45^

To reduce emissions from hospitalizations, efforts should focus on preventing common complications in CKD, including heart failure, infections, and acute kidney injury.^46^ This requires the appropriate medication use and patient education on preventive strategies. In addition, interventions shown to lower hospital admissions and readmissions in other chronic diseases should be trialed in CKD, such as caregiver involvement in care plans,^47^ comprehensive discharge planning,^48^ and optimized medication management^49^ to reduce medication-related adverse events.

Telemedicine can reduce travel emissions while maintaining care quality and patient satisfaction.^50^ Sensitivity analyses suggest telehealth could lower emissions by ≤15% in the UK and 11% in the USA, depending on the CKD stage. For necessary in-person visits, the continued electrification of road transport will play a key role in reducing emissions, alongside active promotion of public transport, walking, cycling, and carpooling, where feasible. Coordinated multidisciplinary clinic days, as trialed by Denmark, reduced hospital visits by 19% and decreased the need for blood sampling by 17%,^51^ offering another model to decrease travel and resource use.

Importantly, CKD stage 5, because of its disproportionately high impact, must be a key target for emissions reduction, especially given the projected increase in kidney failure cases in coming decades.^52^ Although treatment must always be patient-centered, our data suggest that transplantation and supportive care should be prioritized over dialysis, where appropriate. Yet, global access to transplantation remains limited as follows: 63% of low-income countries’ transplantation services^53^ and nearly half (46%) of eligible patients in high income countries do not receive a transplant. Access to conservative care for kidney management is similarly inadequate, currently available in only 53% of countries.^53^ Global initiatives are urgently needed to expand transplantation through the development of supportive legislative and policy frameworks in all countries. Efforts to increase international awareness, availability, and standardization of conservative kidney management is also essential via the establishment of written guidelines, shared decision-making tools, and access to multidisciplinary teams and services.^53^

Consistent with previous data,^20^ our study shows that PD has a lower GHG impact than HD, with CAPD generating less GHG emissions than APD. This supports a “PD first” approach,^54^ prioritizing PD unless contraindicated, and favoring CAPD over APD unless clinical or psychosocial factors dictate otherwise. Progress toward these priorities may be aided by reimbursement policies that incentivize PD^54^ alongside dialysis services to build expertise and experience in PD patient management.

Although HD is well-recognized as having a high environmental impact,^55^ progress in mitigating this has been slow.^56^ National policies and regulatory changes should establish and enforce stringent environmental standards, mandate environmental reporting, and incentivize technological innovation and sustainable practices. Educating and supporting clinical staff is crucial. Empowering staff through leadership opportunities, such as “Sustainability Champions” within dialysis units can promote grassroots change and help embed sustainable practices.^57^ Visible institutional support, including alignment of sustainability with departmental goals and the inclusion of green initiatives in professional recognition structures, can further reinforce engagement.^58^ In addition, global collaboration and advocacy, such as through the ISN’s "GREEN-K" (Global Environmental Evolution in Nephrology and Kidney Care) initiative,^59^ will be essential for accelerating progress across regions.

We evaluated environmental impacts of CKD beyond GHG emissions. Similar to GHG emissions, these increased with disease progression across all categories. Importantly, many pollutants, such as fine particulate matter (i.e., PM_2.5_) negatively impact both the environment and public health, affecting cardiovascular, respiratory, renal, neurological, gastrointestinal, and reproductive systems.^60^ The total CKD population generated 54,700 and 1260 tonnes PM_2.5_ emissions/yr in the USA and UK, respectively. We estimate that health damages from this pollution would equate to 34,600 and 799 disability-adjusted life years lost in the 2 countries, respectively, highlighting the broader consequences of pollution linked to health care delivery.

In our study, the difference in GHG emissions between the USA and the UK is noteworthy, though not unexpected. The USA health care system is recognized as one of the most carbon-intense in the world.^61^ In contrast, the UK National Health Service, supported by a favorable policy and regulatory environment, has committed to achieving net-zero health care emissions by 2040 and made significant progress toward this goal.^62^ Similarly, the UK kidney care sector has established ambitious targets for both decarbonization and the reduction of other environmental impacts, setting an example for others to follow.

Our exploratory analyses involving Australia, Belgium, Brazil, Germany, Japan, Italy, Netherlands, and Spain, revealed up to 2.3-fold variation in annual per-person emissions, attributable to differences in energy sources; travel distances; as well as heating, cooling, and lighting requirements between countries. Importantly, all these countries lacked comprehensive datasets on health care resource use, which necessitated the use of UK and USA proxy data. To better design and implement country-specific strategies for reducing the environmental impact of CKD care, detailed health care datasets are needed.

Our study has several limitations. Limited information on proprietary processes and materials created uncertainty in some model inputs. Medications were a particular area of uncertainty because of scarce environmental impact data from most manufacturers, the wide range of prescriptions, and variable prescribing practices.^63^ Because medications were excluded from our main analyses and some patients with CKD may take up to 27 different medications a day,^63^ our results likely underestimate the true GHG emissions impact of CKD. Comprehensive LCA data for pharmaceuticals, particularly those kidney protective pharmaceuticals are called for.^64^ Although the DISCOVER CKD datasets provide real-world health care resource use data, they do not distinguish whether each health care encounter is directly attributable to CKD. Given the interconnected nature of CKD and its sequelae, we deemed it appropriate to capture all health care resource use, because these interactions collectively represent the real-world care pathway.^39^^,^^40^ For the KRT population, hospitalization impacts may have been underestimated, because inputs were extrapolated from data from patients receiving supportive care, whose illnesses are typically less intensively investigated and treated compared with dialysis and transplant patients. In contrast, the impact of transport may be overestimated because of assumptions made regarding the proportions of patients using personal transportation versus hospital or public transport. This is particularly relevant in countries like the UK, where government-funded hospital transport allows patients to travel together, thereby reducing the impact. We also only considered 1 treatment regimen for each dialysis modality. Different impacts would be expected with alternative regimens. For example, home HD could be performed for 4 hours thrice weekly instead of 8 hours, or for 8 hours nightly. PD might involve lower fill volumes (e.g., 1.5–2.0 L instead of 2.5 L) or fewer exchanges/day, as commonly observed in clinical practice.^65^ Similarly, the environmental impact of newer home HD technologies which may be associated with reduced GHG emissions was not addressed in the present study. Results rely on secondary data and may not be generalizable to individual dialysis services. To ensure the development of optimally targeted mitigation strategies, local data collection and validation of our results are required. It should be reiterated that the estimates do not capture the environmental impact of undiagnosed CKD, which may be substantial but is far more difficult to quantify given the lack of visibility in health care datasets. Therefore, our results represent a conservative, lower-bound estimate of the true population-level burden, focusing only on those patients identified and managed within health care systems.

In conclusion, CKD imposes a high environmental burden, adding to its substantial clinical, humanistic, and economic impacts. This burden intensifies as the disease progresses, peaking with KRT. Dialysis, hospitalizations, and transportation were identified as key hotspots, with KRT contributing disproportionately to total emissions despite its relatively small share of the CKD population. These findings provide a foundation for targeted strategies to reduce the environmental impact of CKD care and support the development of more sustainable care models.

## Disclosure

KAB, AC, RP-F, CG-D, CZ, and MJE received consulting fees for taking part in Scientific Steering Committee meetings regarding CKD environmental impact research. AC has received research grants from CSL Vifor; consultancy fees from Astellas, AstraZeneca, Bayer, Boehringer Ingelheim, GSK, Lilly, Novo Nordisk, Otsuka, CSL Vifor; and lecture fees from Astellas, AstraZeneca, Amgen, Bayer, Medscape, Novo Nordisk, Sanofi (Mexico), and CSL Vifor. RP-F has received research grants from Fresenius Medical Care and the National Council for Scientific and Technological Development; grants (paid to employer) from AstraZeneca, Boehringer-Lilly, Novo Nordisk, Akebia, and Bayer for participation in advisory boards and educational activities. RP-F is employed by Arbor Research Collaborative for Health, which runs the DOPPS studies. Global support for the ongoing DOPPS Programs is provided without restriction on publications by a variety of funders. Funding is provided to Arbor Research Collaborative for Health and not to RPF directly. JJGS, VS, SB, and CC are employees and shareholders of AstraZeneca. AW, NE, LH, and LN received consulting fees from AstraZeneca.

## Supplementary Material

Supplementary File (PDF) **Supplementary Methods. Supplementary References. Figure S1.** Exploratory analyses show that as CKD progresses, carbon emissions increase, with at-home and in-center HD being the largest contributors in the disease pathway per patient. **Table S1.** Overview of common Life Cycle Impact Assessment methods and their applications. **Table S2.** Proportions of patients receiving different kidney replacement therapy modalities at CKD stage 5, based on published registry data and Inside-CKD data. **Table S3.** Inside-CKD prevalence data. **Table S4.** Health care resource use data used in the model for the USA. **Table S5.** Health care resource use data used in the model for the UK. **Table S6.** CKD stage-specific HCRU and associated GHG emissions used for the USA. **Table S7.** CKD stage-specific HCRU and associated GHG emissions used for the UK. **Table S8.** Estimated GHG emissions (tonnes CO_2_e) and percentage contribution by health care resource for the CKD population in the USA and UK. **Table S9.** Environmental impact∗ increases as CKD progresses in the USA per patient. **Table S10.** Environmental impact∗ increases as CKD progresses in the UK per patient. **Table S11.** Environmental impact∗ was greatest at CKD stage 3 in the US prevalent population. **Table S12.** Environmental impact∗ was greatest at CKD stage 3 in the UK prevalent population. **Table S13.** Exploratory analyses: GHG emissions per population. **Table S14.** Sensitivity analysis showed how altering the different model parameters in the US affected annual GHG emissions per population at each CKD stage. **Table S15.** Sensitivity analysis showed how altering the different model parameters in the UK affected annual GHG emissions per population at each CKD stage. **Table S16.** Environmental impact of CKD: a summary of the key findings, implications, and recommended actions. STROBE checklist.
