# Supplementary material for: International Environmental Impact of CKD Care
Source: Kidney Int Rep. 2025 Oct 30;11(2):103662. doi: 10.1016/j.ekir.2025.10.019 (PMC12805029; doi:10.1016/j.ekir.2025.10.019)
Supplement: Supplementary File (PDF) — Supplementary Methods. Supplementary References. Figure S1. Exploratory analyses show that as CKD progresses, carbon emissions increase, with at-home and in-center HD being the largest contributors in the disease pathway per patient. Table S1. Overview of common Life Cycle Impact Assessment methods and their applications. Table S2. Proportions of patients receiving different kidney replacement therapy modalities at CKD stage 5, based on published registry data and Inside-CKD data. Table S3. Inside-CKD prevalence data. Table S4. Health care resource use data used in the model for the USA. Table S5. Health care resource use data used in the model for the UK. Table S6. CKD stage-specific HCRU and associated GHG emissions used for the USA. Table S7. CKD stage-specific HCRU and associated GHG emissions used for the UK. Table S8. Estimated GHG emissions (tonnes CO2e) and percentage contribution by health care resource for the CKD population in the USA and UK. Table S9. Environmental impact∗ increases as CKD progresses in the USA per patient. Table S10. Environmental impact∗ increases as CKD progresses in the UK per patient. Table S11. Environmental impact∗ was greatest at CKD stage 3 in the US prevalent population. Table S12. Environmental impact∗ was greatest at CKD stage 3 in the UK prevalent population. Table S13. Exploratory analyses: GHG emissions per population. Table S14. Sensitivity analysis showed how altering the different model parameters in the US affected annual GHG emissions per population at each CKD stage. Table S15. Sensitivity analysis showed how altering the different model parameters in the UK affected annual GHG emissions per population at each CKD stage. Table S16. Environmental impact of CKD: a summary of the key findings, implications, and recommended actions. STROBE checklist. [file mmc1.docx]

**SUPPLEMENTARY MATERIAL**

**International environmental impact of chronic kidney disease care**

**Corresponding author:**

Juan Jose Garcia Sanchez

AstraZeneca,

Barcelona,

Spain

Email: juanjose.garciasanchez@astrazeneca.com

Katherine A. Barraclough, PhD^1,2^

Aleix Cases, PhD^3^

Matthew J. Eckelman, PhD^4^

Celine Germond-Duret, PhD^5^

Carmine Zoccali^6,7,8^

Nina Embleton, PhD^9^

Antony Wright, MSc^9^

Luke Hubbert, MMath^9^

Lindsay Nicholson, PhD^9^

Salvatore Barone, PharmD^10^

Claudia Cabrera, PhD^11^

Juan Jose Garcia Sanchez, MSc^12^

Viknesh Selvarajah, PhD^13^

Roberto Pecoits-Filho, PhD^14,15^

^1^Department of Nephrology, Royal Melbourne Hospital, Australia

^2^Department of Medicine, University of Melbourne, Parkville, Victoria, Australia

^3^Nephrology Unit, Hospital Clínic de Barcelona, Spain

^4^Department of Civil & Environmental Engineering, Northeastern University, Boston, USA

^5^School of Global Affairs, Lancaster University, UK

^6^Renal Research Institute, New York, USA

^7^Institute of Biology and Molecular Genetics, Ariano Irpino, Italy

^8^Associazione Ipertensione Nefrologia Trapianto Renale (IPNET), c/o Nefrologia, Grande Ospedale Metropolitano, Reggio Calabria, Italy

^9^Maverex Limited, Newcastle upon Tyne, UK

^10^Global Medical Affairs, AstraZeneca, Boston, USA

^11^Emerging Medicines, BioPharmaceutical Medical AstraZeneca, Gothenburg, Sweden

^12^Global Market Access & Pricing, AstraZeneca, Barcelona, Spain

^13^Research and Early Development, Cardiovascular, Renal and Metabolism, BioPharmaceuticals R&D, AstraZeneca, Cambridge, UK

^14^Clin Epi Program Area, Arbor Research Collaborative for Health, Ann Arbor, MI USA

^15^Department of Medicine, Pontificia Universidade Catolica do Parana, Curitiba, Brazil

**Corresponding author:**

Juan Jose Garcia Sanchez

AstraZeneca,

Barcelona,

Spain

Email: juanjose.garciasanchez@astrazeneca.com

**STROBE Statement—checklist of items that should be included in reports of observational studies**

|  | Item No. | Recommendation | Page  No. | Relevant text from manuscript |
| --- | --- | --- | --- | --- |
| **Title and abstract** | 1 | (*a*) Indicate the study’s design with a commonly used term in the title or the abstract | Page 1 | The environmental impact of chronic kidney disease: results of an international life cycle assessment |
|  |  |  | Page 3 | We performed a life cycle assessment of the environmental impact of CKD stages 1–5, with a primary focus on greenhouse gas (GHG) emissions and a secondary aim of quantifying broader environmental effects |
|  |  | (*b*) Provide in the abstract an informative and balanced summary of what was done and what was found | Page 3 | We performed a life cycle assessment of CKD stages 1–5, with a primary focus on greenhouse gas (GHG) emissions and a secondary aim of quantifying broader environmental effects. The main scope estimated annual environmental impacts in the US and UK, both per patient and for the total CKD population, with eight additional countries included in exploratory analyses. Model inputs (annual healthcare resource use, travel distance, energy mix, heating/cooling/lighting requirements) were country-specific, where available. Environmental impacts by stage were calculated using the ReCiPe impact assessment method.  In the US and UK, annual per patient GHG emissions increased with CKD stage, from 1.9–7.8 tonnes and 0.4–5.1 tonnes of carbon dioxide equivalents (CO2e), respectively, with similar trends for other environmental impacts. Total annual GHG emissions were 30.6 and 1.8 megatonnes CO2e in the US and UK, respectively, with stage 3 contributing the greatest proportion. Hospitalisation drove emissions for stages 1–4, for stage 5 on supportive care, and for the prevalent transplant population. For patients receiving kidney replacement therapy, choice of modality drove GHG emissions. Although only 4.7% of the US CKD population and 1.1% of the UK population received KRT, this accounted for 14% and 8% of national CKD emissions, respectively, largely from thrice-weekly in-centre haemodialysis. |
| Introduction | | | |  |
| Background/rationale | 2 | Explain the scientific background and rationale for the investigation being reported | Page 4 | While data exists on the environmental impact of specific aspects of kidney care, such as the carbon footprint of haemodialysis (HD)^1–6^ and peritoneal dialysis (PD),^5,7^ there is a paucity of data on the environmental impact of the broader CKD care pathway, including comparative data on the different CKD stages and kidney replacement therapy (KRT) modalities. |
| Objectives | 3 | State specific objectives, including any prespecified hypotheses | Page 4 | The primary aim of this study was to identify GHG emissions and 'hotspots' across all CKD stages and KRT modalities, and to compare countries to highlight best practices in delivering low-carbon CKD care. A secondary aim of the analysis was to quantify wider environmental impacts. |
| Methods | | | |  |
| Study design | 4 | Present key elements of study design early in the paper | Page 5 | Key elements presented in the study overview paragraph, lines 124–141 |
| Setting | 5 | Describe the setting, locations, and relevant dates, including periods of recruitment, exposure, follow-up, and data collection | Page 5 | Data collection described on page 5, data collected from literature sources. Primary data was not collected in this study |
| Participants | 6 | (*a*) *Cohort study*—Give the eligibility criteria, and the sources and methods of selection of participants. Describe methods of follow-up  *Case-control study*—Give the eligibility criteria, and the sources and methods of case ascertainment and control selection. Give the rationale for the choice of cases and controls  *Cross-sectional study*—Give the eligibility criteria, and the sources and methods of selection of participants | N/A |  |
|  |  | (*b*) *Cohort study*—For matched studies, give matching criteria and number of exposed and unexposed  *Case-control study*—For matched studies, give matching criteria and the number of controls per case | N/A |  |
| Variables | 7 | Clearly define all outcomes, exposures, predictors, potential confounders, and effect modifiers. Give diagnostic criteria, if applicable | N/A |  |
| Data sources/ measurement | 8* | For each variable of interest, give sources of data and details of methods of assessment (measurement). Describe comparability of assessment methods if there is more than one group | N/A |  |
| Bias | 9 | Describe any efforts to address potential sources of bias | N/A |  |
| Study size | 10 | Explain how the study size was arrived at | N/A |  |

Continued on next page

| Quantitative variables | | 11 | | Explain how quantitative variables were handled in the analyses. If applicable, describe which groupings were chosen and why | N/A |  | |
| --- | --- | --- | --- | --- | --- | --- | --- |
| Statistical methods | | 12 | | (*a*) Describe all statistical methods, including those used to control for confounding | N/A |  | |
|  |  |  |  | (*b*) Describe any methods used to examine subgroups and interactions | N/A |  | |
|  |  |  |  | (*c*) Explain how missing data were addressed | N/A |  | |
|  |  |  |  | (*d*) *Cohort study*—If applicable, explain how loss to follow-up was addressed  *Case-control study*—If applicable, explain how matching of cases and controls was addressed  *Cross-sectional study*—If applicable, describe analytical methods taking account of sampling strategy | N/A |  | |
|  |  |  |  | (*e*) Describe any sensitivity analyses | Page 6 | The robustness of the results was assessed using one-way sensitivity analyses, varying key parameters individually to examine their impact on overall environmental outcomes. Parameters tested included assumptions around telemedicine and electric transport use, distances travelled, heating/cooling/lighting values to reflect different climates, the number and length of hospitalisation events, and inclusion of medications. This approach highlighted the variables with the greatest influence on results. | |
| Results | | | | | | | |
| Participants | | 13* | | (a) Report numbers of individuals at each stage of study—eg numbers potentially eligible, examined for eligibility, confirmed eligible, included in the study, completing follow-up, and analysed | N/A |  | |
|  |  |  |  | (b) Give reasons for non-participation at each stage | N/A |  | |
|  |  |  |  | (c) Consider use of a flow diagram | N/A |  | |
| Descriptive data | | 14* | | (a) Give characteristics of study participants (eg demographic, clinical, social) and information on exposures and potential confounders | N/A |  | |
|  |  |  |  | (b) Indicate number of participants with missing data for each variable of interest | N/A |  | |
|  |  |  |  | (c) *Cohort study*—Summarise follow-up time (eg, average and total amount) | N/A |  | |
| Outcome data | | 15* | | *Cohort study*—Report numbers of outcome events or summary measures over time | N/A |  | |
|  |  |  |  | *Case-control study—*Report numbers in each exposure category, or summary measures of exposure | N/A |  | |
|  |  |  |  | *Cross-sectional study—*Report numbers of outcome events or summary measures | N/A |  | |
| Main results | | 16 | | (*a*) Give unadjusted estimates and, if applicable, confounder-adjusted estimates and their precision (eg, 95% confidence interval). Make clear which confounders were adjusted for and why they were included | N/A |  | |
|  |  |  |  | (*b*) Report category boundaries when continuous variables were categorized | N/A |  | |
|  |  |  |  | (*c*) If relevant, consider translating estimates of relative risk into absolute risk for a meaningful time period | N/A |  | |
| Other analyses | 17 | | Report other analyses done—eg analyses of subgroups and interactions, and sensitivity analyses | | Page 8 | | Incorporating telemedicine into the care pathway for 50% of GP and specialist care visits led to a 2─10% reduction in per patient GHG emissions in the US and a 1─15% reduction in the UK, compared with baseline emissions, depending on CKD stage. Tables 4 and 5 detail the reductions in GHG emissions observed from switching a proportion of petrol vehicles to electric vehicles, optimising heating, cooling, and lighting requirements, and reducing the healthcare stays and health visits. When including the impact of medications in analyses, GHG emissions increased by up to 151% in the US and 172% in the UK. However, these increases should be interpreted with caution, as modelling was based on limited available data. |
| Discussion | | | | | | | |
| Key results | 18 | | Summarise key results with reference to study objectives | | Page 8-11 | | See discussion text |
| Limitations | 19 | | Discuss limitations of the study, taking into account sources of potential bias or imprecision. Discuss both direction and magnitude of any potential bias | | Page 11 | | Our study has several limitations. Limited information on proprietary processes and materials created uncertainty in some model inputs. Medications were a particular area of uncertainty due to scarce environmental impact data from most manufacturers, the wide range of prescriptions, and variable prescribing practices.^62^ Since medications were excluded from our main analyses and some patients with CKD may take up to 27 different medications a day,^62^ our results likely underestimate the true GHG emissions impact of CKD. Comprehensive LCA data for pharmaceuticals, particularly those kidney protective pharmaceuticals are called for.^63^ While the DISCOVER CKD datasets provide real-world healthcare resource utilisation data, they do not distinguish whether each healthcare encounter is directly attributable to CKD. Given the interconnected nature of CKD and its sequelae, we deemed it appropriate to capture all healthcare resource use, as these interactions collectively represent the real-world care pathway.^38,39^ For the KRT population, hospitalisation impacts may have been underestimated, as inputs were extrapolated from data from patients receiving supportive care, whose illnesses are typically less intensively investigated and treated compared with dialysis and transplant patients. In contrast, the impact of transport may be overestimated due to assumptions made regarding the proportions of patients using personal transportation versus hospital or public transport. This is particularly relevant in countries like the UK, where government-funded hospital transport allows patients to travel together, thereby reducing the impact. We also only considered one treatment regimen for each dialysis modality. Different impacts would be expected with alternative regimens. For example, home HD could be performed for four hours thrice weekly instead of eight hours, or for eight hours nightly. PD might involve lower fill volumes (e.g., 1.5–2.0 L instead of 2.5 L) or fewer exchanges per day, as commonly observed in clinical practice.^64^ Similarly, the environmental impact of newer home haemodialysis technologies which may be associated with reduced GHG emissions was not addressed in the present study. Results rely on secondary data and may not be generalisable to individual dialysis services. To ensure the development of optimally targeted mitigation strategies, local data collection and validation of our results are required. It should also be reiterated that the estimates do not capture the environmental impact of undiagnosed CKD, which may be substantial but is far more difficult to quantify given the lack of visibility in healthcare datasets. As such, our results represent a conservative, lower-bound estimate of the true population-level burden, focusing only on those patients identified and managed within healthcare systems. |
| Interpretation | 20 | | Give a cautious overall interpretation of results considering objectives, limitations, multiplicity of analyses, results from similar studies, and other relevant evidence | | Page 8-11 | |  |
| Generalisability | 21 | | Discuss the generalisability (external validity) of the study results | |  | |  |
| Other information | | |  | | | | |
| Funding | 22 | | Give the source of funding and the role of the funders for the present study and, if applicable, for the original study on which the present article is based | | Page 12 | | AstraZeneca funded this study |

**SUPPLEMENTARY METHODS**

**Databases and software**

The CKD disease treatment pathway was modelled using LCA for experts (formerly GaBi) software (v10.7.1.28), with inputs from the ecoinvent Life Cycle Inventory database version 3.8.^29^ Environmental impact categories were calculated using ReCiPe 2016 v1.1 impact assessment methodology.^22^

**Data collection**

Scope

A life cycle analysis (LCA) was developed to include all disease stages of CKD, over ten countries: the United Kingdom (UK), and the United States of America (USA), Australia, Belgium, Brazil, Germany, Japan, Italy, the Netherlands and Spain.

Data collection

A targeted literature review was conducted to understand the care pathway for patients with CKD, define the study boundary, to gather inputs for each module, and to determine country-specific modelling parameters (such as average length of healthcare visit, transport time, and dialysis parameters).

Scientific Steering Committee

A scientific steering committee (SSC) was formed to advise on the design, implementation, interpretation, and validation of the environmental impact model. Members belonged to a range of relevant fields including nephrology and environmental science, including those with clinical experience of CKD. The SSC advised on the modelling approach and clinical assumptions, reviewed gaps in country-specific data and use of proxies, and aided interpretation of results from a global and country-specific perspective.

Data gaps and proxy data

Where country-specific inputs were lacking in the ecoinvent database for materials, energy, and emissions relating to any aspect of the CKD treatment pathway, proxy data was used to match the production characteristics, as detailed previously.^24^ Use of proxy data was reviewed and validated with the SSC to ensure data was representative.

**Life cycle inventory analysis**

The LCA was conducted according to ISO 14040/14044 international standards.

Each CKD disease stage was built from several life cycle stages to cover the whole care pathway of a patient with CKD. Life cycle stages included primary care visits; specialist care visits; intensive care unit visits; emergency room visits; hospitalisations; haemodialysis (HD); peritoneal dialysis (PD); and kidney transplant surgery (and work up).

Each life cycle stage was built using its component inputs, including heating/lighting/cooling for a defined length of appointment/stay, materials/consumables, waste disposal, and patient travel. Full details for each life cycle stage are published elsewhere^24^ and are described briefly here. Healthcare resource use data was then applied to build the CKD care pathway at each disease stage. The study boundary outlines what was included and excluded from the model (Figure 2).

**Modelling life cycle stages in the CKD care pathway**

Primary care visits

A GP visit was comprised of the transport required to attend the location, and the heating/cooling/lighting of the clinic, proportioned to the scale of one patient over 30 minutes (10 minutes for the consultation, 20 minutes waiting time).^S1^ Additional electricity usage not associated with heating/cooling/lighting was not included and was assumed to be negligible.^S2^ Blood and urine tests were excluded due to lack of resource use data.

Specialist care visits

Specialist care refers to CKD-related outpatient care that includes, but is not limited to, nephrology consultations. It encompasses care provided by a range of specialists such as endocrinologists, cardiologists, and dietitians, particularly in early stages of CKD. This reflects the multidisciplinary nature of CKD management, given the strong clinical overlap with comorbid conditions like diabetes and hypertension. Specialist care visits were modelled as per a GP visit, with the following amendments. Distance travelled to the specialist clinic was increased. Duration of appointment including waiting time was increased to four hours to account for blood tests, blood pressure assessment/weight monitoring and going to the pharmacy, in addition to the specialist visit.

Intensive care unit visit

A previous study evaluating the carbon footprint of patients treated in an intensive care unit (ICU) in the USA and Australia for septic shock was used as input for this module.^S2^ As one of the most common causes of ICU admission for patients with CKD is sepsis,^S3^ this study was deemed appropriate. Inputs for ICU include heating/cooling/lighting, additional drug mix (morphine, anaesthetic, iron sulfate, saline), hospital waste disposal, washing and laundry, intravenous (IV) drip, cotton, steel, plastics, oxygen, paper, rubber, and card. Full details of the ICU inputs have been previously published.^24^

Hospital waste was split between incineration, landfill, and recycling. The heating/cooling/lighting was proportioned to the scale of one patient for their length of stay.

An ICU stay was assumed to be followed by a hospitalisation.

Hospitalisation

Hospitalisation was modelled similarly to the ICU, with removal of the additional drug mix and oxygen, and the remaining inputs decreased by 25% to reflect its less intensive nature, validated with the SSC for relevancy. When modelling different CKD disease stages, the same hospital module was used for each, with the option to adjust length of stay.

Emergency room visit

The emergency room (ER) visit was based on a hospital visit, but with a shorter duration of eight hours. After visiting the ER, a patient was assumed to either go on to an ICU or hospital stay, with proportions based on the actual ratio of hospital/ICU stages per year for each CKD stage in each country.^38,39^

Haemodialysis

HD was assumed to require a dialysis machine (requiring water, dialysate, and electricity), heating/cooling/lighting for the area of treatment, consumables (cloth, packaging, dialyser, tubing, etc.) waste disposal, transport, and the initial vascular access surgery. At-home and in-centre HD differed in the amount of heating/cooling/lighting required, based on different areas and energy requirements. Both were assumed to use the same quantity of consumables. Waste disposal proportions were modelled to be the same as a hospitalisation. Transport of the electrolyte mix were included but had low impact, estimated using a weighted average distance from one distribution centre to the most populated cities in the UK. Key parameters modelled for HD have been previously published.^24^

Peritoneal dialysis

Two methods of PD were modelled, automated PD (APD) and continuous ambulatory PD (CAPD). APD was assumed to involve a machine, heating/cooling/lighting for the area of treatment, the peritoneal fluid and drainage bags, and associated packaging/tubing. Exchanges were modelled as glucose exchanges (modelled as PD exchanges with glucose as osmotic agent). Transport of the peritoneal fluid was included but had a low impact. CAPD was modelled as per APD but without machinery, as this is not required for dialysate exchanges. Energy consumption for the surgical placement of PD catheter was also included.

Key parameters used for PD have been previously published.^24^

Kidney transplant surgery (and work up)

Both living and deceased donors were modelled as a source of kidney transplantation. Both were assumed to have similar inputs, with differences including the number of scans, hospital stays, and transportation of the organ (deceased donor only). For the transplant recipient, modelling included workup scans, day stays in hospital (pre- and post-transplant) and the transplant surgery itself. The operation was assumed to use the following materials: glass, steel, plastic, paper/card, and cotton. Anaesthetics for undergoing surgery and Marshall’s hypertonic citrate were included for organ transportation. As with the modules, transport and heating/cooling/lighting of the building where the surgery takes place were included. To account for delayed graft function, it was assumed that 30% of the recipients receiving a kidney from a deceased donor would require haemodialysis, of which three sessions were included, modelled as in-centre HD, without transport.

Key parameters have been previously published.^24^

Energy mix

The kWh/m^2^ energy requirement for heating/cooling/lighting of each healthcare area (ICU/hospital, clinics, laboratory/operating room or at-home) was obtained by literature review,^33–37^ with proxy data from the UK used where country-specific data were lacking.^36^ Hospital energy requirements were modelled based on results from the ICU with a reduction of 25% as an assumption based on the reduced intensity of care.

These values were then scaled to express energy requirement per patient in each healthcare area, with the m^2^ per patient based on specifications derived from the UK Department of Health.^30–32^ Assumed total areas per patient have been previously published.^24^

Per patient per-area energy requirements were then scaled to reflect the energy mix of each country, sourced from ecoinvent Life Cycle Inventory database v3.8.15.^29^ This describes the proportion of electricity vs natural gas used by each country. Together, these values defined the overall environmental impact due to heating/cooling/lighting for each life cycle stage.

Patient transport

Travel was modelled for module each based on published methodology.^S4^ Modes of transportation (car/bus/train/ambulance/walking) varied based on CKD stage and destination, with the proportions for each stage and mode of travel published elsewhere.^24^ Calculated travel times were converted to distances, with average speed varying based on distance to destination (for example, when within 10 minutes of a healthcare facility, speed would be that for residential roads). Where facility co-ordinates were unavailable, an approximation based on another country was used, as noted. Collection of medication from pharmacy was included.

Medication

The average environmental impact of the five most common types of drugs prescribed to patients with CKD was calculated:^38^

- Renin-angiotensin-aldosterone system (RAAS) inhibitors (captopril)
- Diuretics (furosemide)
- Anticoagulants (warfarin)
- Antiplatelet agents (aspirin)
- Anti-hypertensive therapies (propranolol beta blocker)

Other common drugs used in CKD such as glucagon-like peptide-1 (GLP-1) receptor agonists or sodium-glucose cotransporter 2 (SGLT2) Inhibitors were not modelled due to lack of propriety information on their impact.

The impact of each drug was assumed to result from five key factors: the active ingredient, the inactive ingredients,^S5,S6^ the average forming process,^S7,S8^ the packaging (blister pack,^S9^ polyethylene terephthalate (PET) bottle^S10,S11^) and transport. The active ingredients were based on the prescribing information for each of the drugs and produced based on publicly available patents, using the original patent values, and the assumption of 90% solvent recycling. Transport of medication was included using an arbitrary distance of 1,000 km by freight lorry.

Full details of the medication inventory analysis have been published elsewhere.^24^

**
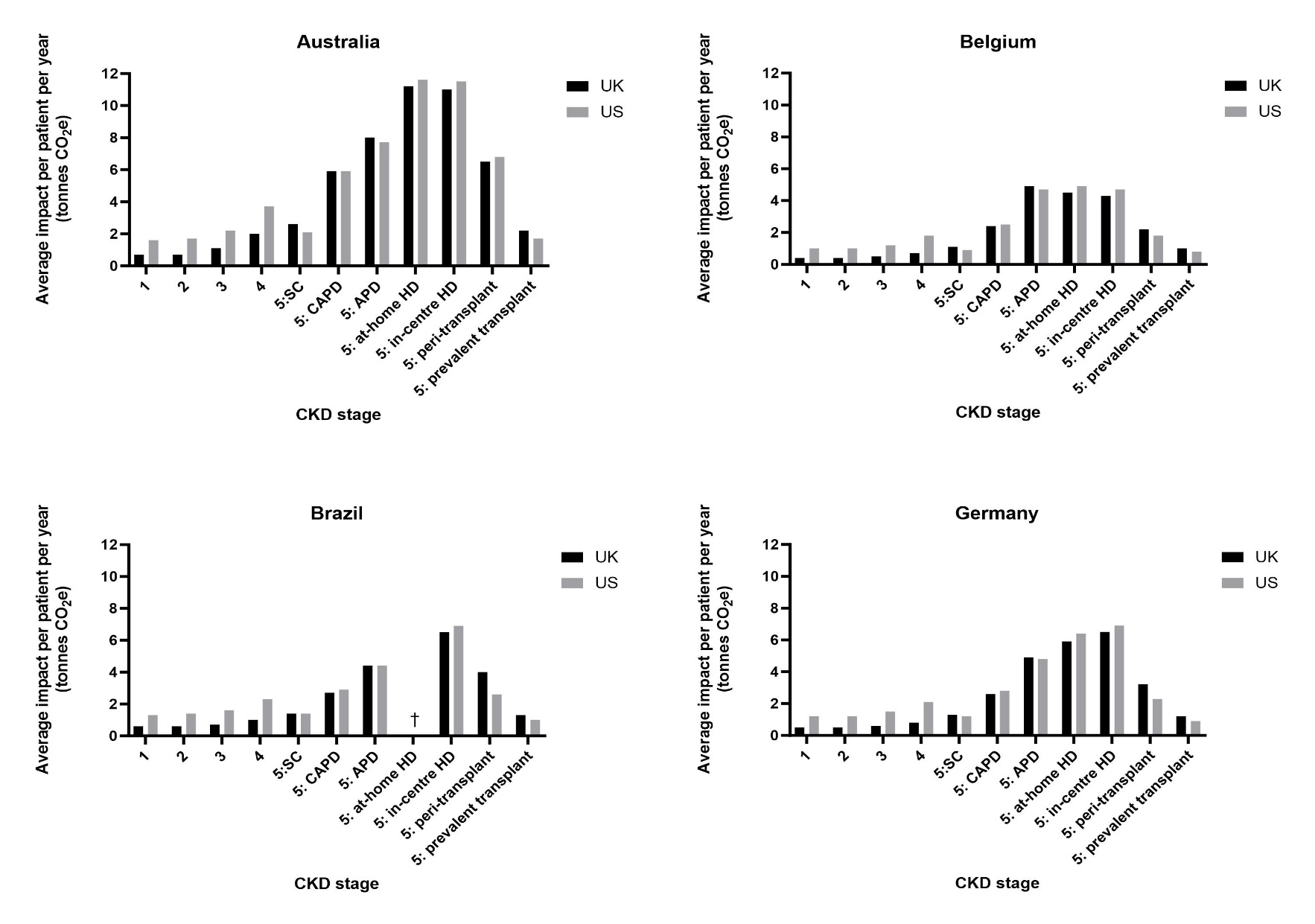
Supplementary Figure 1.** **Exploratory analyses* show that as CKD progresses, carbon emissions increase, with at-home and in-centre HD the largest contributors in the disease pathway per patient**

**
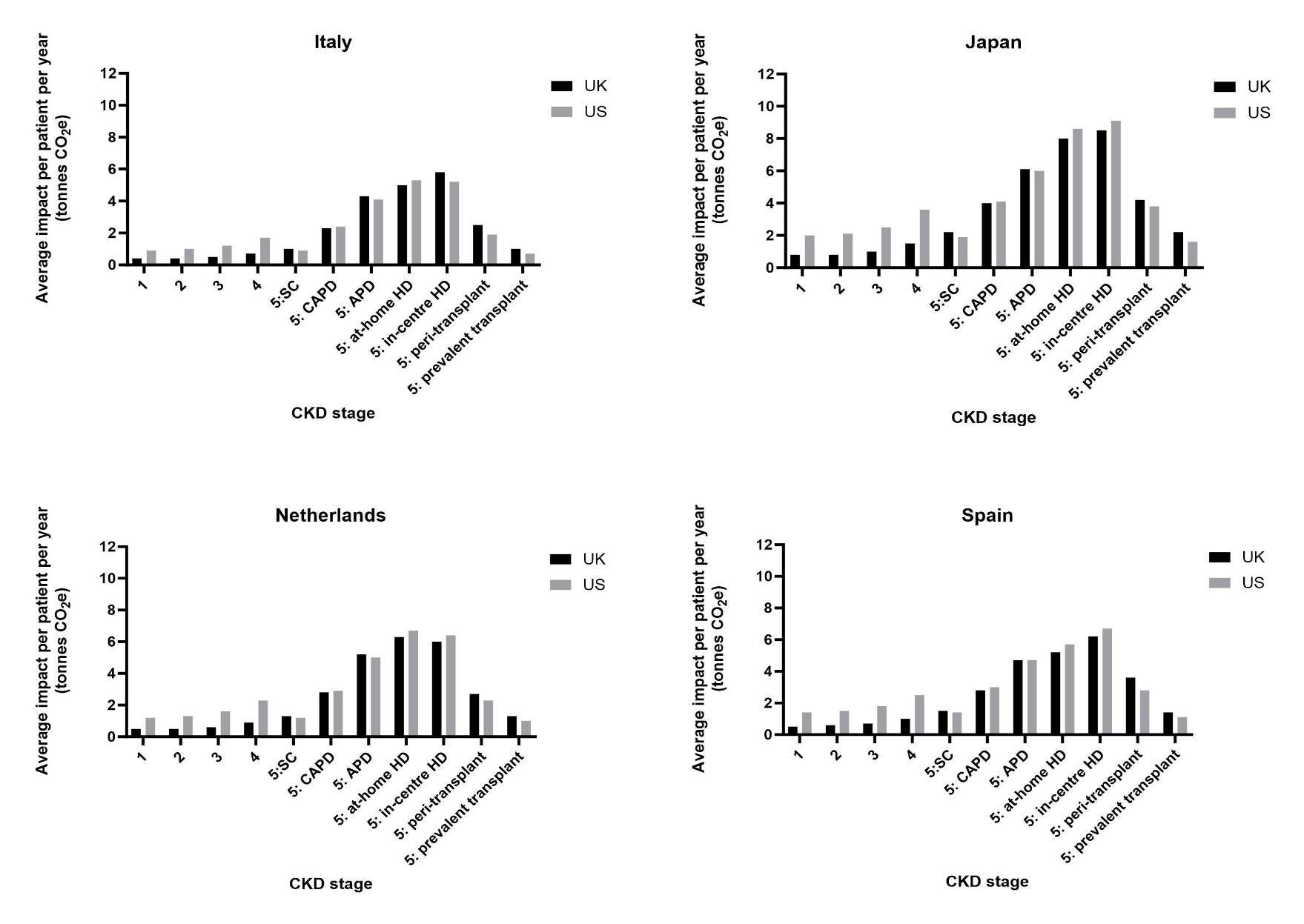
Supplementary Figure 1 (cont’d). Exploratory analyses* show that as CKD progresses, carbon emissions increase, with at-home and in-centre HD the largest contributors in the disease pathway per patient**

*Due to absence of data, healthcare resource use data was used from UK (black bars) and USA datasets (grey bars); †No home HD conducted in Brazil. APD, automated peritoneal dialysis; CAPD, continuous ambulatory peritoneal dialysis; CKD, chronic kidney disease; CO_2_e, carbon dioxide equivalents; HD, haemodialysis; SC, supportive care; UK, United Kingdom; US, United States.

| **Method, Reference** | **Full Name** | **Developed By** | **Description** | **Common Impact Categories** |
| --- | --- | --- | --- | --- |
| **CML**^S12^ | Centre of Environmental Science of Leiden University Method | Leiden University (Netherlands) | A midpoint-oriented LCIA method that provides a set of indicators for environmental impact based on problem-oriented (midpoint) approaches. Widely used in European studies. | Global warming, acidification, eutrophication, photochemical oxidation, ozone depletion, abiotic resource depletion |
| **ReCiPe**^S13^ | "ReCiPe" stands for Resource use and Emissions Cause Environmental Impacts | Developed jointly by Dutch institutes (RIVM, Radboud University, PRé Consultants) | Integrates midpoint and endpoint modelling to connect emissions to both environmental mechanisms and potential damages to human health and ecosystems. | Global warming, fine particulate matter formation, human toxicity, land use, water consumption, fossil resource use |
| **TRACI^23^** | Tool for the Reduction and Assessment of Chemical and Other Environmental Impacts | U.S. Environmental Protection Agency | Designed specifically for U.S. conditions, TRACI enables LCIA with region-specific data and characterisation factors. | Global warming, acidification, eutrophication, ozone depletion, smog formation, human health cancer and non-cancer effects |

**Supplementary Table 1. Overview of common Life Cycle Impact Assessment methods and their applications**

CML, Centre of Environmental Science of Leiden University Method; LCIA, Life Cycle Impact Assessment; ReCiPe, Resource use and Emissions Cause Environmental Impacts; RIVM, Rijksinstituut voor Volksgezondheid en Milieu; TRACI, Tool for the Reduction and Assessment of Chemical and Other Environmental Impacts; U.S. EPA, United States Environmental Protection Agency.,

**Supplementary Table 2. Proportions of patients receiving different kidney replacement therapy modalities at CKD stage 5, based on published registry data and Inside-CKD data^25^**

|  | **Australia^S14^** | **Belgium^†^** | **Brazil^S15^** | **Germany^28^** | **Italy^†^** | **Japan^S16^** | **Netherlands^S17^** | **Spain^S17^** | **UK^27^** | **USA^S18^** |
| --- | --- | --- | --- | --- | --- | --- | --- | --- | --- | --- |
| **5: SC*** | 0.015 | 0.015 | 0.015 | 0.015 | 0.015 | 0.015 | 0.015 | 0.015 | 0.015 | 0.015 |
| **5: Dialysis** | 0.507 | 0.529 | 0.736 | 0.712 | 0.592 | 0.951 | 0.321 | 0.463 | 0.402 | 0.688 |
| **5: CAPD** | 0.054 | 0.030 | 0.014 | 0.030 | 0.03 | 0.011 | 0.065 | 0.137 | 0.077 | 0.095 |
| **5: APD** | 0.121 | 0.030 | 0.054 | 0.030 | 0.03 | 0.011 | 0.084 | 0.040 | 0.051 | 0.015 |
| **5: at-home HD** | 0.076 | 0.007 | 0.000 | 0.007 | 0.007 | 0.002 | 0.045 | 0.002 | 0.047 | 0.022 |
| **5: in-centre HD** | 0.749 | 0.934 | 0.932 | 0.934 | 0.934 | 0.976 | 0.806 | 0.823 | 0.825 | 0.869 |
| **5: peri-transplant** | 0.010 | 0.012 | 0.003 | 0.001 | 0.022 | 0.000 | 0.004 | 0.005 | 0.008 | 0.003 |
| **5: prevalent transplant** | 0.468 | 0.444 | 0.246 | 0.271 | 0.371 | 0.034 | 0.661 | 0.517 | 0.575 | 0.295 |

*Percentage of patients at CKD stage 5 receiving supportive care not widely collected by registry studies, it was assumed that 1.5% of patients would be receiving supportive care based on data reported by the UK Renal Registry (2022); †German data were used due to lack of country-specific data.

APD, automated peritoneal dialysis; CAPD, continuous ambulatory peritoneal dialysis; CKD, chronic kidney disease; HD, haemodialysis; SC, supportive care; UK, United Kingdom; US, United States of America.

**Supplementary Table 3. Inside-CKD prevalence data^25^**

| **CKD stage** | **Australia** | **Belgium** | **Brazil** | **Germany** | **Italy** | **Japan** | **Netherlands** | **Spain** | **UK** | **USA** |
| --- | --- | --- | --- | --- | --- | --- | --- | --- | --- | --- |
| **1** | 40,773 | 15,211 | 148,413 | 298,795 | 53,229 | 121,284 | 23,686 | 34,913 | 89,840 | 787,520 |
| **2** | 152,988 | 75,289 | 996,229 | 371,852 | 200,257 | 2,087,532 | 126,794 | 230,485 | 424,959 | 3,364,606 |
| **3** | 309,444 | 345,923 | 4,995,639 | 856,733 | 690,145 | 4,771,983 | 404,381 | 1,289,979 | 1,962,918 | 6,833,335 |
| **4** | 25,124 | 61,261 | 357,945 | 66,076 | 91,485 | 534,646 | 27,837 | 82,280 | 173,038 | 425,353 |
| **5: SC** | 424 | 230 | 3,216 | 1,386 | 1,240 | 5,586 | 294 | 934 | 1,092 | 12,484 |
| **5: CAPD*** | 778 | 627 | 2,150 | 1,942 | 3,779 | 3,894 | 407 | 3,950 | 2,258 | 54,445 |
| **5: APD*** | 1,731 | 415 | 8,601 | 1,942 | 2,502 | 3,894 | 528 | 1,153 | 1,496 | 8,473 |
| **5: at-home HD*** | 1,091 | 383 | 0 | 486 | 2,306 | 708 | 286 | 58 | 1,378 | 12,366 |
| **5: in-centre HD*** | 10,752 | 6,700 | 147,081 | 61,455 | 40,386 | 345,483 | 5,071 | 23,726 | 24,134 | 497,221 |
| **5: peri-transplant** | 270 | 187 | 637 | 130 | 1,812 | 171 | 74 | 315 | 561 | 2,144 |
| **5: prevalent transplant** | 13,239 | 6,816 | 52,722 | 25,091 | 30,688 | 12,648 | 12,961 | 32,183 | 41,902 | 245,131 |

*Proportioned according to registry data (see Supplementary Table 2), where available.

APD, automated peritoneal dialysis; CAPD, continuous ambulatory peritoneal dialysis; CKD, chronic kidney disease; HD, haemodialysis; SC, supportive care; UK, United Kingdom; US, United States of America.

**Supplementary Table 4. Healthcare resource use data used in the model for the USA**

|  | **Rate per person-year** | | | | | | | | |
| --- | --- | --- | --- | --- | --- | --- | --- | --- | --- |
|  | **CKD stage 1** | **CKD stage 2** | **CKD stage 3** | **CKD stage 4** | **CKD stage 5:**  **Supportive care** | **CKD stage 5:**  **Haemodialysis** | **CKD stage 5:**  **Peritoneal dialysis** | **CKD stage 5:**  **Peri-transplant** | **CKD stage 5:**  **Prevalent transplant** |
| Annual primary care visits per patient | 5.1^S19^ | 4.5^S19^ | 5.4^S19^ | 5.1^S19^ | 5.6^S19^ | 4.5^S20^ | DISCOVER-CKD for CKD 5 used (4.5)^39^ | DISCOVER-CKD for CKD 5 used (4.5)^39^ | DISCOVER-CKD for CKD 5 used (4.5)^39^ |
| Annual specialist care visits per patient | 10.1^39*^ | 11.3^39*^ | 12.9^39*^ | 15.2^39*^ | 11.8^39*^ | DISCOVER-CKD for CKD 5 used (11.8)^39^ | DISCOVER-CKD for CKD 5 used (11.8)^39^ | 13.6^S21^ | 4.4^S21^ |
| Annual emergency department visits per patient | 0.1^39*^ | 0.2^39*^ | 0.2^39*^ | 0.3^39*^ | 0.3^39*^ | 1.5^S18^ | 1.1^S18^ | 1.6^S21^ | 1.0^S21^ |
| Annual intensive care visits per patient (+ LOS) | 0.3 (NR)^39*^ | 0.3 (NR)^39*^ | 0.3 (NR)^39*^ | 0.7 (NR)^39*^ | 0.3 (NR)^39*^ | 0.2 (4.3)^S22**^ | Strijack et al 2009 used as proxy (0.2)^S22^ | 0.2 (NR)^S23^ | Abrol et al (2019) used as proxy (0.2)^S23^ |
| Annual hospitalisations per patient (+ LOS) | 2.0 (NR)^39*^ | 2.0 (NR)^39*^ | 2.6 (NR)^39*^ | 3.5 (NR)^39*^ | 1.7 (NR)^39*^ | 1.8 (NR)^18^ | 1.5 (NR)^S18^ | 1.5 (NR)^S18^ | 0.7 (NR)^S18^ |

*Data based on weighted mean across urine-albumin creatinine ratio 0–<30 mg/g, 30–<300 mg/g, and ≥300 mg/g; ** data based on Canadian study population.

CKD, chronic kidney disease; LOS, length of stay; NR, length of stay not reported; USA, United States of America.

**Supplementary Table 5. Healthcare resource use data used in the model for the UK**

|  | **Rate per person-year** | | | | | | | | |
| --- | --- | --- | --- | --- | --- | --- | --- | --- | --- |
|  | **CKD stage 1** | **CKD stage 2** | **CKD stage 3** | **CKD stage 4** | **CKD stage 5:**  **Supportive care** | **CKD stage 5:**  **Haemodialysis** | **CKD stage 5:**  **Peritoneal dialysis** | **CKD stage 5:**  **Peri-transplant** | **CKD stage 5:**  **Prevalent transplant** |
| Annual primary care visits per patient | 11.3^38*^ | 11.3^38*^ | 12.3^38*^ | 12.6^38*^ | 13.6^38*^ | DISCOVER-CKD for CKD 5 used (13.6)^38*^ | DISCOVER-CKD for CKD 5 used (13.6)^38*^ | DISCOVER-CKD for CKD 5 used (13.6)^38*^ | DISCOVER-CKD for CKD 5 used (13.6)^38*^ |
| Annual specialist care visits per patient | 2.8^38*^ | 2.8^38*^ | 3.1^38*^ | 3.9^38*^ | 5.9^38*^ | 4^S24**^  Used as proxy | 4^S24**^  Used as proxy | 40.0^S24**^ | 4.0^S24**^ |
| Annual emergency department visits per patient | 0.3^38*^ | 0.3^38*^ | 0.5^38*^ | 0.6^38*^ | 0.7^38*^ | DISCOVER-CKD for CKD 5 used (0.7)^38*^ | DISCOVER-CKD for CKD 5 used (0.7)^38*^ | DISCOVER-CKD for CKD 5 used (0.7) ^38*^ | DISCOVER-CKD for CKD 5 used (0.7)^38*^ |
| Annual intensive care visits per patient (+ LOS) | 0.01 (NR)^38*^ | 0.01 (NR)^38*^ | 0.02 (NR)^38*^ | 0.02 (NR)^38**^ | 0.03 (NR)^38*^ | 0.06 (1.9)^S25^ | Hutchison et al (2007) used (0.06)^S25^ | 0.05 (3.7–18)^S26^ | DISCOVER-CKD for CKD 5 used (0.03)^38*^ |
| Annual hospitalisations per patient (+ LOS) | 0.5 (NR)^38*^ | 0.6 (14.7)^38*^ | 0.7 (NR)^38*^ | 1.1 (NR)^38*^ | 2.0 (54.3)^38*^ | DISCOVER-CKD for CKD 5 used (2.0)^38*^ | DISCOVER-CKD for CKD 5 used (2.0)^38*^ | DISCOVER-CKD for CKD 5 used (2.0)^38*^ | DISCOVER-CKD for CKD 5 used  (2.0)^38*^ |

*Data based on weighted mean across urine-albumin creatinine ratio 0-<30 mg/g, 30-<300 mg/g, and ≥300 mg/g; ** data based on guidelines.

CKD, chronic kidney disease; LOS, length of stay; UK, United Kingdom.

**Supplementary Table 6: CKD stage-specific HCRU and associated GHG emissions used for the USA**

| **Stage** | **Input** | **Unit** | **Quantity used** | **Emission factor (kg CO₂e/unit)** | **Total emissions per patient (kg CO₂e)** |
| --- | --- | --- | --- | --- | --- |
| **1** | GP Visit | per visit | 5.10 | 0.30 | 1.55 |
|  | Specialist Care Visit | per visit | 10.10 | 2.36 | 23.83 |
|  | ER visit | per visit | 0.10 | 17.45 | 1.74 |
|  | Hospitalisation | per bed day | 26.17 | 52.33 | 1369.67 |
|  | ICU stay | per bed day | 1.31 | 124.68 | 163.93 |
|  | Transport: |  |  |  |  |
|  | to GP | return transport (km) | 5.10 | 6.85 | 34.92 |
|  | to specialist | return transport (km) | 10.10 | 21.64 | 218.59 |
|  | to ER | return transport (km) | 0.10 | 6.56 | 0.66 |
|  | to hospital | return transport (km) | 2.00 | 6.22 | 12.43 |
|  | to ICU | return transport (km) | 0.30 | 6.87 | 2.06 |
|  | to pick up medication | return transport (km) | 12.00 | 3.79 | 45.47 |
| **2** | GP Visit | per visit | 4.50 | 0.30 | 1.37 |
|  | Specialist Care Visit | per visit | 11.30 | 2.36 | 26.66 |
|  | ER visit | per visit | 0.20 | 17.45 | 3.49 |
|  | Hospitalisation | per bed day | 27.27 | 52.33 | 1426.74 |
|  | ICU stay | per bed day | 1.37 | 124.68 | 170.76 |
|  | Transport: |  |  |  |  |
|  | to GP | return transport (km) | 4.50 | 6.85 | 30.81 |
|  | to specialist | return transport (km) | 11.30 | 21.64 | 244.56 |
|  | to ER | return transport (km) | 0.20 | 6.82 | 1.36 |
|  | to hospital | return transport (km) | 2.00 | 6.31 | 12.63 |
|  | to ICU | return transport (km) | 0.30 | 7.33 | 2.20 |
|  | to pick up medication | return transport (km) | 12.00 | 3.79 | 45.47 |
| **3** | GP Visit | per visit | 5.40 | 0.30 | 1.64 |
|  | Specialist Care Visit | per visit | 12.90 | 2.36 | 30.44 |
|  | ER visit | per visit | 0.20 | 17.45 | 3.49 |
|  | Hospitalisation | per bed day | 34.45 | 52.33 | 1802.92 |
|  | ICU stay | per bed day | 1.35 | 124.68 | 167.94 |
|  | Transport: |  |  |  |  |
|  | to GP | return transport (km) | 5.40 | 6.85 | 36.98 |
|  | to specialist | return transport (km) | 12.90 | 21.64 | 279.19 |
|  | to ER | return transport (km) | 0.20 | 7.11 | 1.42 |
|  | to hospital | return transport (km) | 2.60 | 6.43 | 16.73 |
|  | to ICU | return transport (km) | 0.30 | 7.79 | 2.34 |
|  | to pick up medication | return transport (km) | 12.00 | 3.79 | 45.47 |
| **4** | GP Visit | per visit | 5.10 | 0.30 | 1.55 |
|  | Specialist Care Visit | per visit | 15.20 | 2.36 | 35.86 |
|  | ER visit | per visit | 0.30 | 17.45 | 5.23 |
|  | Hospitalisation | per bed day | 47.82 | 52.33 | 2502.33 |
|  | ICU stay | per bed day | 3.15 | 124.68 | 392.75 |
|  | Transport: |  |  |  |  |
|  | to GP | return transport (km) | 5.10 | 8.37 | 42.70 |
|  | to specialist | return transport (km) | 15.20 | 24.21 | 368.06 |
|  | to ER | return transport (km) | 0.30 | 7.75 | 2.32 |
|  | to hospital | return transport (km) | 3.50 | 7.24 | 25.33 |
|  | to ICU | return transport (km) | 0.70 | 8.26 | 5.78 |
|  | to pick up medication | return transport (km) | 12.00 | 4.63 | 55.59 |
| **5: SC** | GP Visit | per visit | 5.60 | 0.30 | 1.70 |
|  | Specialist Care Visit | per visit | 11.80 | 2.36 | 27.84 |
|  | ER visit | per visit | 0.30 | 17.45 | 5.23 |
|  | Hospitalisation | per bed day | 24.74 | 52.33 | 1294.44 |
|  | ICU stay | per bed day | 0.00 | 124.68 | 0.00 |
|  | Transport: |  |  |  |  |
|  | to GP | return transport (km) | 5.60 | 9.92 | 55.57 |
|  | to specialist | return transport (km) | 11.80 | 24.66 | 290.97 |
|  | to ER | return transport (km) | 0.30 | 8.64 | 2.59 |
|  | to hospital | return transport (km) | 1.70 | 8.13 | 13.83 |
|  | to ICU | return transport (km) | 0.00 | 9.15 | 0.00 |
|  | to pick up medication | return transport (km) | 12.00 | 5.49 | 65.89 |
| **5: CAPD** | GP Visit | per visit | 4.50 | 0.30 | 1.37 |
|  | Specialist Care Visit | per visit | 11.80 | 2.36 | 27.84 |
|  | ER visit | per visit | 1.10 | 17.45 | 19.19 |
|  | Hospitalisation | per bed day | 30.81 | 52.33 | 1612.40 |
|  | ICU stay | per bed day | 1.38 | 124.68 | 172.50 |
|  | CAPD | per day | 365.00 | 4.47 | 1631.79 |
|  | Transport: |  |  |  |  |
|  | to GP | return transport (km) | 4.50 | 9.39 | 42.25 |
|  | to specialist | return transport (km) | 11.80 | 24.66 | 290.97 |
|  | to ER | return transport (km) | 1.10 | 8.38 | 9.21 |
|  | to hospital | return transport (km) | 1.50 | 8.04 | 12.06 |
|  | to ICU | return transport (km) | 0.20 | 8.69 | 1.74 |
|  | to pick up medication | return transport (km) | 12.00 | 5.19 | 62.34 |
| **5: APD** | GP Visit | per visit | 4.50 | 0.30 | 1.37 |
|  | Specialist Care Visit | per visit | 11.80 | 2.36 | 27.84 |
|  | ER visit | per visit | 1.10 | 17.45 | 19.19 |
|  | Hospitalisation | per bed day | 30.81 | 52.33 | 1612.40 |
|  | ICU stay | per bed day | 1.38 | 124.68 | 172.50 |
|  | APD | per day | 365 | 9.20 | 3358.25 |
|  | Transport: |  |  |  |  |
|  | to GP | return transport (km) | 4.50 | 9.39 | 42.25 |
|  | to specialist | return transport (km) | 11.80 | 24.66 | 290.97 |
|  | to ER | return transport (km) | 1.10 | 8.38 | 9.21 |
|  | to hospital | return transport (km) | 1.50 | 8.04 | 12.06 |
|  | to ICU | return transport (km) | 0.20 | 8.69 | 1.74 |
|  | to pick up medication | return transport (km) | 12.00 | 5.19 | 62.34 |
| **5: Home HD** | GP Visit | per visit | 4.50 | 0.30 | 1.37 |
|  | Specialist Care Visit | per visit | 11.80 | 2.36 | 27.84 |
|  | ER visit | per visit | 1.50 | 17.45 | 26.17 |
|  | Hospitalisation | per bed day | 38.99 | 52.33 | 2040.42 |
|  | ICU stay | per bed day | 1.47 | 124.68 | 183.29 |
|  | At-home HD | per session | 208 | 23.96 | 4983.53 |
|  | Transport: |  |  |  |  |
|  | to GP | return transport (km) | 4.50 | 9.39 | 42.25 |
|  | to specialist | return transport (km) | 11.80 | 24.66 | 290.97 |
|  | to ER | return transport (km) | 1.50 | 8.38 | 12.57 |
|  | to hospital | return transport (km) | 1.80 | 8.04 | 14.47 |
|  | to ICU | return transport (km) | 0.20 | 8.69 | 1.74 |
|  | to pick up medication | return transport (km) | 12.00 | 5.19 | 62.34 |
| **5: In-centre HD** | GP Visit | per visit | 4.50 | 0.30 | 1.37 |
|  | Specialist Care Visit | per visit | 11.80 | 2.36 | 27.84 |
|  | ER visit | per visit | 1.50 | 17.45 | 26.17 |
|  | Hospitalisation | per bed day | 38.99 | 52.33 | 2040.42 |
|  | ICU stay | per bed day | 1.47 | 124.68 | 183.29 |
|  | In-centre HD | per session | 156 | 25.51 | 3979.33 |
|  | Transport: |  |  |  |  |
|  | to GP | return transport (km) | 4.50 | 9.39 | 42.25 |
|  | to specialist | return transport (km) | 11.80 | 24.66 | 290.97 |
|  | to ER | return transport (km) | 1.50 | 8.38 | 12.57 |
|  | to hospital | return transport (km) | 1.80 | 8.04 | 14.47 |
|  | to ICU | return transport (km) | 0.20 | 8.69 | 1.74 |
|  | to pick up medication | return transport (km) | 12.00 | 5.19 | 62.34 |
|  | to HD | return transport (km) | 156.00 | 6.77 | 1152.84 |
| **5: Peritransplant** | GP Visit | per visit | 4.50 | 0.30 | 1.37 |
|  | Specialist Care Visit | per visit | 13.60 | 2.36 | 32.09 |
|  | ER visit | per visit | 1.60 | 17.45 | 27.92 |
|  | Hospitalisation | per bed day | 36.95 | 52.33 | 1933.36 |
|  | ICU stay | per bed day | 2.13 | 124.68 | 265.20 |
|  | Transplant (living donor)* | per transplant | 0.413 | 746.18 | 308.17 |
|  | Transplant (deceased donor) | per transplant | 0.587 | 2026.52 | 1189.57 |
|  | In-centre HD | per session | 0.528 | 25.51 | 13.50 |
|  | Transport: |  |  |  |  |
|  | to GP | return transport (km) | 4.50 | 9.39 | 42.25 |
|  | to specialist | return transport (km) | 13.60 | 24.66 | 335.35 |
|  | to ER | return transport (km) | 1.60 | 8.38 | 13.40 |
|  | to hospital | return transport (km) | 1.50 | 8.04 | 12.06 |
|  | to ICU | return transport (km) | 0.20 | 8.69 | 1.74 |
|  | to pick up medication | return transport (km) | 12.00 | 5.19 | 62.34 |
| **5: Prevalent transplant** | GP Visit | per visit | 4.50 | 0.30 | 1.37 |
|  | Specialist Care Visit | per visit | 4.40 | 2.36 | 10.38 |
|  | ER visit | per visit | 1.00 | 17.45 | 17.45 |
|  | Hospitalisation | per bed day | 19.38 | 52.33 | 1013.94 |
|  | ICU stay | per bed day | 1.77 | 124.68 | 221.11 |
|  | Transport: |  |  |  |  |
|  | to GP | return transport (km) | 4.50 | 9.39 | 42.25 |
|  | to specialist | return transport (km) | 4.40 | 24.66 | 108.50 |
|  | to ER | return transport (km) | 1.00 | 8.38 | 8.38 |
|  | to hospital | return transport (km) | 0.70 | 8.04 | 5.63 |
|  | to ICU | return transport (km) | 0.20 | 8.69 | 1.74 |
|  | to pick up medication | return transport (km) | 12.00 | 5.19 | 62.34 |

*Transplant (living donor) module includes scans, hospital stays, donor and recipient surgery (including anaesthetics), and patient transport. Deceased donor module additionally includes marshall's hypertonic citrate for organ transportation.

APD, automated peritoneal dialysis; CAPD, continuous ambulatory peritoneal dialysis; CO_2_e, carbon dioxide equivalents; GP, general practitioner; ER, emergency room; HD, haemodialysis; ICU, intensive care unit; SC, supportive care; USA, United States of America.

**Supplementary Table 7. CKD stage-specific HCRU and associated GHG emissions used for the UK**

| **CKD stage** | **Input** | **Unit** | **Quantity Used** | **Emission factor (kg CO₂e/unit)** | **Total emissions per patient (kg CO₂e)** |
| --- | --- | --- | --- | --- | --- |
| **1** | GP Visit | per visit | 11.30 | 0.12 | 1.32 |
|  | Specialist Care Visit | per visit | 2.80 | 0.91 | 2.54 |
|  | ER visit | per visit | 0.30 | 10.31 | 3.09 |
|  | Hospitalisation | per bed day | 9.53 | 30.91 | 294.49 |
|  | ICU stay | per bed day | 0.07 | 77.24 | 5.15 |
|  | Transport: |  |  |  |  |
|  | to GP | return transport (km) | 11.30 | 3.81 | 43.00 |
|  | to specialist | return transport (km) | 2.80 | 15.59 | 43.64 |
|  | to ER | return transport (km) | 0.30 | 4.72 | 1.42 |
|  | to hospital | return transport (km) | 0.50 | 4.48 | 2.24 |
|  | to ICU | return transport (km) | 0.01 | 4.95 | 0.05 |
|  | to pick up medication | return transport (km) | 12.00 | 3.79 | 45.47 |
| **2** | GP Visit | per visit | 11.30 | 0.12 | 1.32 |
|  | Specialist Care Visit | per visit | 2.80 | 0.91 | 2.54 |
|  | ER visit | per visit | 0.30 | 10.31 | 3.09 |
|  | Hospitalisation | per bed day | 10.72 | 30.91 | 331.55 |
|  | ICU stay | per bed day | 0.06 | 77.24 | 4.84 |
|  | Transport: |  |  |  |  |
|  | to GP | return transport (km) | 11.30 | 3.81 | 43.00 |
|  | to specialist | return transport (km) | 2.80 | 15.59 | 43.64 |
|  | to ER | return transport (km) | 0.30 | 4.91 | 1.47 |
|  | to hospital | return transport (km) | 0.60 | 4.55 | 2.73 |
|  | to ICU | return transport (km) | 0.01 | 5.28 | 0.05 |
|  | to pick up medication | return transport (km) | 12.00 | 3.79 | 45.47 |
| **3** | GP Visit | per visit | 12.30 | 0.12 | 1.44 |
|  | Specialist Care Visit | per visit | 3.10 | 0.91 | 2.82 |
|  | ER visit | per visit | 0.50 | 10.31 | 5.15 |
|  | Hospitalisation | per bed day | 14.27 | 30.91 | 441.18 |
|  | ICU stay | per bed day | 0.14 | 77.24 | 10.99 |
|  | Transport: |  |  |  |  |
|  | to GP | return transport (km) | 12.30 | 3.81 | 46.80 |
|  | to specialist | return transport (km) | 3.10 | 15.59 | 48.32 |
|  | to ER | return transport (km) | 0.50 | 5.12 | 2.56 |
|  | to hospital | return transport (km) | 0.70 | 4.63 | 3.24 |
|  | to ICU | return transport (km) | 0.02 | 5.61 | 0.11 |
|  | to pick up medication | return transport (km) | 12.00 | 3.79 | 45.47 |
| **4** | GP Visit | per visit | 12.60 | 0.12 | 1.47 |
|  | Specialist Care Visit | per visit | 3.90 | 0.91 | 3.54 |
|  | ER visit | per visit | 0.60 | 10.31 | 6.19 |
|  | Hospitalisation | per bed day | 20.25 | 30.91 | 626.06 |
|  | ICU stay | per bed day | 0.13 | 77.24 | 9.96 |
|  | Transport: |  |  |  |  |
|  | to GP | return transport (km) | 12.60 | 4.65 | 58.62 |
|  | to specialist | return transport (km) | 3.90 | 17.44 | 68.01 |
|  | to ER | return transport (km) | 0.60 | 5.58 | 3.35 |
|  | to hospital | return transport (km) | 1.10 | 5.21 | 5.73 |
|  | to ICU | return transport (km) | 0.02 | 5.95 | 0.12 |
|  | to pick up medication | return transport (km) | 12.00 | 4.63 | 55.59 |
| **5: SC** | GP Visit | per visit | 13.60 | 0.12 | 1.59 |
|  | Specialist Care Visit | per visit | 5.90 | 0.91 | 5.36 |
|  | ER visit | per visit | 0.70 | 10.31 | 7.22 |
|  | Hospitalisation | per bed day | 32.21 | 30.91 | 995.69 |
|  | ICU stay | per bed day | 0.00 | 77.24 | 0.00 |
|  | Transport: |  |  |  |  |
|  | to GP | return transport (km) | 13.60 | 5.51 | 75.00 |
|  | to specialist | return transport (km) | 5.90 | 17.76 | 104.77 |
|  | to ER | return transport (km) | 0.70 | 6.23 | 4.36 |
|  | to hospital | return transport (km) | 2.00 | 5.86 | 11.72 |
|  | to ICU | return transport (km) | 0.00 | 6.59 | 0.00 |
|  | to pick up medication | return transport (km) | 12.00 | 5.49 | 65.89 |
| **5: CAPD** | GP Visit | per visit | 13.60 | 0.12 | 1.59 |
|  | Specialist Care Visit | per visit | 4.00 | 0.91 | 3.64 |
|  | ER visit | per visit | 0.70 | 10.31 | 7.22 |
|  | Hospitalisation | per bed day | 32.26 | 30.91 | 997.19 |
|  | ICU stay | per bed day | 0.34 | 77.24 | 26.08 |
|  | CAPD | per day | 365 | 3.63 | 1323.59 |
|  | Transport: |  |  |  |  |
|  | to GP | return transport (km) | 13.60 | 5.22 | 70.95 |
|  | to specialist | return transport (km) | 4.00 | 17.76 | 71.03 |
|  | to ER | return transport (km) | 0.70 | 6.03 | 4.22 |
|  | to hospital | return transport (km) | 2.00 | 5.79 | 11.58 |
|  | to ICU | return transport (km) | 0.06 | 6.26 | 0.38 |
|  | to pick up medication | return transport (km) | 12.00 | 5.19 | 62.34 |
| **5: APD** | GP Visit | per visit | 13.60 | 0.12 | 1.59 |
|  | Specialist Care Visit | per visit | 4.00 | 0.91 | 3.64 |
|  | ER visit | per visit | 0.70 | 10.31 | 7.22 |
|  | Hospitalisation | per bed day | 32.26 | 30.91 | 997.19 |
|  | ICU stay | per bed day | 0.34 | 77.24 | 26.08 |
|  | APD | per day | 365 | 8.46 | 3088.13 |
|  | Transport: |  |  |  |  |
|  | to GP | return transport (km) | 13.60 | 5.22 | 70.95 |
|  | to specialist | return transport (km) | 4.00 | 17.76 | 71.03 |
|  | to ER | return transport (km) | 0.70 | 6.03 | 4.22 |
|  | to hospital | return transport (km) | 2.00 | 5.79 | 11.58 |
|  | to ICU | return transport (km) | 0.06 | 6.26 | 0.38 |
|  | to pick up medication | return transport (km) | 12.00 | 5.19 | 62.34 |
| **5: Home HD** | GP Visit | per visit | 13.60 | 0.12 | 1.59 |
|  | Specialist Care Visit | per visit | 4.00 | 0.91 | 3.64 |
|  | ER visit | per 8 hr visit | 0.70 | 10.31 | 7.22 |
|  | Hospitalisation | per bed day | 32.26 | 30.91 | 997.19 |
|  | ICU stay | per bed day | 0.34 | 77.24 | 26.08 |
|  | At-home HD | per day | 208 | 17.77 | 3695.70 |
|  | Transport: |  |  |  |  |
|  | to GP | return transport (km) | 13.60 | 5.22 | 70.95 |
|  | to specialist | return transport (km) | 4.00 | 17.76 | 71.03 |
|  | to ER | return transport (km) | 0.70 | 6.03 | 4.22 |
|  | to hospital | return transport (km) | 2.00 | 5.79 | 11.58 |
|  | to ICU | return transport (km) | 0.06 | 6.26 | 0.38 |
|  | to pick up medication | return transport (km) | 12.00 | 5.19 | 62.34 |
| **5: In-centre HD** | GP Visit | per visit | 13.60 | 0.12 | 1.59 |
|  | Specialist Care Visit | per visit | 4.00 | 0.91 | 3.64 |
|  | ER visit | per visit | 0.70 | 10.31 | 7.22 |
|  | Hospitalisation | per bed day | 32.26 | 30.91 | 997.19 |
|  | ICU stay | per bed day | 0.34 | 77.24 | 26.08 |
|  | In-centre HD | per session | 156 | 15.71 | 2450.79 |
|  | Transport: |  |  |  |  |
|  | to GP | return transport (km) | 13.60 | 5.22 | 70.95 |
|  | to specialist | return transport (km) | 4.00 | 17.76 | 71.03 |
|  | to ER | return transport (km) | 0.70 | 6.03 | 4.22 |
|  | to hospital | return transport (km) | 2.00 | 5.79 | 11.58 |
|  | to ICU | return transport (km) | 0.06 | 6.26 | 0.38 |
|  | to pick up medication | return transport (km) | 12.00 | 5.19 | 62.34 |
|  | to HD | return transport (km) | 156.00 | 9.11 | 1421.16 |
| **5: Peritransplant** | GP Visit | per visit | 13.60 | 0.12 | 1.59 |
|  | Specialist Care Visit | per visit | 40.00 | 0.91 | 36.35 |
|  | ER visit | per visit | 0.70 | 10.31 | 7.22 |
|  | Hospitalisation | per bed day | 32.87 | 30.91 | 1016.20 |
|  | ICU stay | per bed day | 0.78 | 77.24 | 60.10 |
|  | Transplant (living donor)* | per transplant | 0.294 | 443.86 | 130.49 |
|  | Transplant (deceased donor) | per transplant | 0.706 | 725.72 | 512.36 |
|  | In-centre HD | per session | 0.635 | 15.71 | 9.98 |
|  | Transport: |  |  |  |  |
|  | to GP | return transport (km) | 13.60 | 5.22 | 70.95 |
|  | to specialist | return transport (km) | 40.00 | 17.76 | 710.30 |
|  | to ER | return transport (km) | 0.70 | 6.03 | 4.22 |
|  | to hospital | return transport (km) | 2.00 | 5.79 | 11.58 |
|  | to ICU | return transport (km) | 0.05 | 6.26 | 0.31 |
|  | to pick up medication | return transport (km) | 12.00 | 5.19 | 62.34 |
| **5: Prevalent transplant** | GP Visit | per visit | 13.60 | 0.12 | 1.59 |
|  | Specialist Care Visit | per visit | 4.00 | 0.91 | 3.64 |
|  | ER visit | per visit | 0.70 | 10.31 | 7.22 |
|  | Hospitalisation | per bed day | 32.21 | 30.91 | 995.69 |
|  | ICU stay | per bed day | 0.17 | 77.24 | 13.09 |
|  | Transport: |  |  |  |  |
|  | to GP | return transport (km) | 13.60 | 5.22 | 70.95 |
|  | to specialist | return transport (km) | 4.00 | 17.76 | 71.03 |
|  | to ER | return transport (km) | 0.70 | 6.03 | 4.22 |
|  | to hospital | return transport (km) | 2.00 | 5.79 | 11.58 |
|  | to ICU | return transport (km) | 0.03 | 6.26 | 0.19 |
|  | to pick up medication | return transport (km) | 12.00 | 5.19 | 62.34 |

*Transplant (living donor) module includes scans, hospital stays, donor and recipient surgery (including anaesthetics), and patient transport. Deceased donor module additionally includes marshall's hypertonic citrate for organ transportation.

APD, automated peritoneal dialysis; CAPD, continuous ambulatory peritoneal dialysis; CO_2_e, carbon dioxide equivalents; GP, general practitioner; ER, emergency room; HD, haemodialysis; ICU, intensive care unit; SC, supportive care; United Kingdom.

**Supplementary Table 8. Estimated GHG emissions (tonnes CO₂e) and percentage contribution by healthcare resource for the CKD population in the USA and UK**

| **Healthcare resource** | **GHG emissions (tonnes CO_2_e) (% CO_2_e contribution)** | |
| --- | --- | --- |
|  | **USA** | **UK** |
| **GP visit** | 18,800 (0.06) | 3,870 (0.22) |
| **Specialist clinic visit** | 351,000 (1.15) | 7,740 (0.44) |
| **ER visit** | 58,100 (0.19) | 13,300 (0.75) |
| **Hospital stay** | 20,700,000 (67.76) | 1,210,000 (68.11) |
| **ICU stay** | 2,180,000 (7.14) | 27,200 (1.53) |
| **Transport** | 5,080,000 (16.63) | 442,000 (24.88) |
| **KRT** | 2,160,000 (7.07) | 72,300 (4.07) |
| **CAPD** | 88,800 (0.29) | 2,990 (0.17) |
| **APD** | 28,500 (0.09) | 4,620 (0.26) |
| **at-home HD** | 61,700 (0.20) | 5,100 (0.29) |
| **in-centre HD** | 1,980,000 (6.48) | 59,300 (3.34) |
| **Transplant** | 3,210 (0.01) | 361 (0.02) |

APD, automated peritoneal dialysis; CAPD, continuous ambulatory peritoneal dialysis; CO_2_e, carbon dioxide equivalents; GP, general practitioner; ER, emergency room; HD, haemodialysis; ICU, intensive care unit; SC, supportive care; UK, United Kingdom; USA, United States of America.

**Supplementary Table 9. Environmental impact* increases as CKD progresses in the USA per patient**

|  | **CKD stage** | | | | | | | | | | |
| --- | --- | --- | --- | --- | --- | --- | --- | --- | --- | --- | --- |
|  | **1** | **2** | **3** | **4** | **5: SC** | **5: CAPD** | **5: APD** | **5: at-home HD** | **5: in-centre HD** | **5: peri-transplant** | **5: prevalent transplant** |
| **Climate change, excl biogenic carbon [tonnes CO_2_ e]** | 1.9 | 2.0 | 2.4 | 3.4 | 1.8 | 3.9 | 5.6 | 7.7 | 7.8 | 4.2 | 1.5 |
| **Climate change, incl biogenic carbon [tonnes CO_2_ e]** | 1.9 | 2.0 | 2.4 | 3.5 | 1.8 | 3.4 | 5.0 | 7.9 | 7.9 | 4.3 | 1.5 |
| **Fine particulate matter formation [kg PM_2.5_ eq.]** | 3.3 | 3.5 | 4.2 | 6.2 | 3.0 | 8.6 | 18.2 | 14.6 | 14.8 | 7.0 | 2.7 |
| **Fossil depletion [kg oil eq.]** | 789.0 | 826.0 | 1,010.0 | 1,450.0 | 724.0 | 1,560.0 | 2,290.0 | 3,150.0 | 3,210.0 | 1,710.0 | 630.0 |
| **Freshwater consumption [m^3^]** | 10.8 | 11.3 | 13.9 | 20.2 | 9.5 | 39.5 | 60.4 | 63.5 | 58.1 | 27.6 | 8.8 |
| **Freshwater ecotoxicity [kg 1,4 DB eq.]** | 54.2 | 57.0 | 68.5 | 97.2 | 57.2 | 121.0 | 167.0 | 257.0 | 240.0 | 102.0 | 42.9 |
| **Freshwater eutrophication [kg P eq.]** | 0.8 | 0.9 | 1.1 | 1.6 | 0.7 | 1.9 | 2.5 | 3.9 | 3.9 | 1.6 | 0.7 |
| **Human toxicity, cancer [kg 1,4-DB eq.]** | 92.7 | 97.4 | 117.0 | 168.0 | 91.3 | 217.0 | 306.0 | 519.0 | 479.0 | 187.0 | 74.1 |
| **Human toxicity, non-cancer [kg 1,4-DB eq.]** | 1,900.0 | 1,990.0 | 2,440.0 | 3,510.0 | 1,770.0 | 3,690.0 | 5,290.0 | 8,130.0 | 8,110.0 | 3,650.0 | 1,520.0 |
| **Ionising radiation [kBq Co-60 eq. to air]** | 316.0 | 330.0 | 406.0 | 592.0 | 274.0 | 583.0 | 736.0 | 1,350.0 | 1,400.0 | 611.0 | 254.0 |
| **Land use [annual crop eq.·y]** | 36.4 | 38.2 | 45.7 | 67.5 | 32.5 | 275.0 | 365.0 | 189.0 | 177.0 | 78.5 | 30.1 |
| **Marine ecotoxicity [kg 1,4-DB eq.]** | 67.7 | 71.4 | 85.6 | 121.0 | 71.8 | 152.0 | 215.0 | 324.0 | 301.0 | 127.0 | 53.7 |
| **Marine eutrophication [kg N eq.]** | 0.1 | 0.1 | 0.2 | 0.2 | 0.1 | 0.7 | 1.0 | 1.2 | 1.1 | 0.3 | 0.1 |
| **Metal depletion [kg Cu eq.]** | 3.2 | 3.4 | 4.0 | 5.7 | 3.6 | 9.4 | 14.0 | 18.3 | 16.2 | 6.9 | 2.5 |
| **Photochemical ozone formation, ecosystems [kg NOx eq.]** | 2.8 | 3.0 | 3.6 | 5.0 | 2.7 | 6.8 | 10.7 | 12.1 | 12.5 | 5.6 | 2.1 |
| **Photochemical ozone formation, human health [kg NOx eq.]** | 2.7 | 2.9 | 3.4 | 4.8 | 2.6 | 6.6 | 10.4 | 11.6 | 12.1 | 5.4 | 2.1 |
| **Stratospheric ozone depletion [g CFC-11 eq.]** | 1.3 | 1.4 | 1.7 | 2.4 | 1.2 | 4.0 | 5.4 | 4.6 | 4.5 | 2.7 | 1.0 |
| **Terrestrial acidification [kg SO_2_ eq.]** | 4.0 | 4.2 | 5.1 | 7.4 | 3.8 | 11.7 | 16.9 | 18.1 | 18.0 | 10.1 | 3.2 |
| **Terrestrial ecotoxicity [kg 1,4-DB eq.]** | 3,220.0 | 3,410.0 | 4,020.0 | 5,660.0 | 3,630.0 | 11,500.0 | 22,700.0 | 17,800.0 | 15,600.0 | 6,290.0 | 2,540.0 |


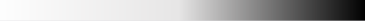


Lowest impact Highest impact

*As reported by ReCiPe 2016 v1.1 Midpoint (H).

APD, automated peritoneal dialysis; CAPD, continuous ambulatory peritoneal dialysis; CFC, chlorofluorocarbon; CKD, chronic kidney disease; Co-60 eq., cobalt-60 equivalent; tonnes CO_2_e, metric tonnes carbon dioxide equivalents; Cu eq., copper equivalent; DB, 1,4 dichlorobenzene; excl, excluding; HD, haemodialysis; incl, including; m^3^, cubic metre; NOx eq., nitrous oxide equivalent; P eq., phosphate equivalent; PM_2.5_, particulate matter, ≤ 2.5 µm; SC, supportive care; SO_2_, sulphur dioxide; US, United States of America.

**Supplementary Table 10. Environmental impact* increases as CKD progresses in the UK per patient**

|  | **CKD stage** | | | | | | | | | | |
| --- | --- | --- | --- | --- | --- | --- | --- | --- | --- | --- | --- |
|  | **1** | **2** | **3** | **4** | **5: SC** | **5: CAPD** | **5: APD** | **5: at-home HD** | **5: in-centre HD** | **5: peri-transplant** | **5: prevalent transplant** |
| **Climate change, excl biogenic carbon [tonnes CO_2_ e]** | 0.4 | 0.5 | 0.6 | 0.8 | 1.3 | 2.6 | 4.3 | 5.0 | 5.1 | 2.6 | 1.2 |
| **Climate change, incl biogenic carbon [tonnes CO_2_ e]** | 0.5 | 0.5 | 0.6 | 0.9 | 1.3 | 2.2 | 3.8 | 5.3 | 5.4 | 2.7 | 1.3 |
| **Fine particulate matter formation [kg PM_2.5_ eq.]** | 0.3 | 0.3 | 0.4 | 0.6 | 0.9 | 2.9 | 5.2 | 4.6 | 4.4 | 2.1 | 0.8 |
| **Fossil depletion [kg oil eq.]** | 192.0 | 210.0 | 271.0 | 375.0 | 574.0 | 1,090.0 | 1,740.0 | 2,340.0 | 2,340.0 | 1,090.0 | 566.0 |
| **Freshwater consumption [m^3^]** | 1.9 | 2.1 | 2.8 | 3.8 | 5.8 | 33.5 | 67.9 | 45.5 | 38.2 | 17.2 | 5.8 |
| **Freshwater ecotoxicity [kg 1,4 DB eq.]** | 16.0 | 17.0 | 20.6 | 28.3 | 42.1 | 88.6 | 134.0 | 203.0 | 193.0 | 93.9 | 39.7 |
| **Freshwater eutrophication [kg P eq.]** | 0.0 | 0.1 | 0.1 | 0.1 | 0.1 | 0.7 | 1.1 | 1.0 | 0.9 | 0.3 | 0.1 |
| **Human toxicity, cancer [kg 1,4-DB eq.]** | 18.8 | 19.9 | 23.9 | 32.7 | 48.1 | 127.0 | 209.0 | 309.0 | 282.0 | 122.0 | 45.2 |
| **Human toxicity, non-cancer [kg 1,4-DB eq.]** | 377.0 | 410.0 | 522.0 | 724.0 | 1,100.0 | 2,100.0 | 3,430.0 | 4,580.0 | 4,470.0 | 2,070.0 | 1,080.0 |
| **Ionising radiation [kBq Co-60 eq. to air]** | 44.5 | 49.4 | 65.5 | 91.2 | 141.0 | 318.0 | 416.0 | 1,220.0 | 1,060.0 | 243.0 | 142.0 |
| **Land use [annual crop eq.·y]** | 10.5 | 11.4 | 14.7 | 20.0 | 29.9 | 263.0 | 351.0 | 241.0 | 213.0 | 71.3 | 29.7 |
| **Marine ecotoxicity [kg 1,4-DB eq.]** | 20.5 | 21.8 | 26.4 | 36.3 | 54.0 | 113.0 | 175.0 | 261.0 | 249.0 | 120.0 | 50.8 |
| **Marine eutrophication [kg N eq.]** | 0.0 | 0.0 | 0.0 | 0.0 | 0.1 | 0.6 | 0.8 | 1.0 | 0.8 | 0.2 | 0.1 |
| **Metal depletion [kg Cu eq.]** | 1.1 | 1.2 | 1.4 | 1.9 | 2.7 | 7.7 | 12.4 | 15.5 | 14.6 | 7.5 | 2.5 |
| **Photochemical ozone formation, ecosystems [kg NOx eq.]** | 0.7 | 0.8 | 0.9 | 1.3 | 1.9 | 4.7 | 8.3 | 9.1 | 9.8 | 4.4 | 1.8 |
| **Photochemical ozone formation, human health [kg NOx eq.]** | 0.7 | 0.7 | 0.9 | 1.2 | 1.8 | 4.6 | 8.1 | 8.8 | 9.4 | 4.3 | 1.7 |
| **Stratospheric ozone depletion [g CFC-11 eq.]** | 0.4 | 0.4 | 0.5 | 0.7 | 1.1 | 3.4 | 4.7 | 3.5 | 3.4 | 2.0 | 1.1 |
| **Terrestrial acidification [kg SO_2_ eq.]** | 0.8 | 0.8 | 1.0 | 1.4 | 2.1 | 7.9 | 12.6 | 11.0 | 10.8 | 5.4 | 2.0 |
| **Terrestrial ecotoxicity [kg 1,4-DB eq.]** | 1,100.0 | 1,150.0 | 1,350.0 | 1,840.0 | 2,680.0 | 9,710.0 | 20,900.0 | 15,600.0 | 14,400.0 | 6,960.0 | 2,460.0 |


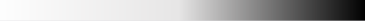


Lowest impact Highest impact

*As reported by ReCiPe 2016 v1.1 Midpoint (H).

APD, automated peritoneal dialysis; CAPD, continuous ambulatory peritoneal dialysis; CFC, chlorofluorocarbon; CKD, chronic kidney disease; Co-60 eq., cobalt-60 equivalent; tonnes CO_2_e, metric tonnes carbon dioxide equivalents; Cu eq., copper equivalent; DB, 1,4 dichlorobenzene; excl, excluding; HD, haemodialysis; incl, including; m^3^, cubic metre; NOx eq., nitrous oxide equivalent; P eq., phosphate equivalent; PM_2.5_, particulate matter, ≤ 2.5 µm; SC, supportive care; SO_2_, sulphur dioxide; UK, United Kingdom.

**Supplementary Table 11. Environmental impact* was greatest at CKD stage 3 in the USA prevalent population**

|  | **CKD stage** | | | | | | | | | | |
| --- | --- | --- | --- | --- | --- | --- | --- | --- | --- | --- | --- |
|  | **1** | **2** | **3** | **4** | **5: SC** | **5: CAPD** | **5: APD** | **5: at-home HD** | **5: in-centre HD** | **5: peri-transplant** | **5: prevalent transplant** |
| **Climate change, excl biogenic carbon [tonnes CO_2_e]** | 1,480,000 | 6,620,000 | 16,300,000 | 1,460,000 | 21,900 | 211,000 | 47,500 | 95,100 | 3,900,000 | 9,080 | 366,000 |
| **Climate change, incl biogenic carbon [tonnes CO_2_e]** | 1,500,000 | 6,710,000 | 16,600,000 | 1,480,000 | 22,300 | 187,000 | 42,600 | 97,100 | 3,950,000 | 9,180 | 371,000 |
| **Fine particulate matter formation [kg PM_2.5_ eq.]** | 2,610,000 | 11,700,000 | 28,900,000 | 2,620,000 | 36,800 | 466,000 | 154,000 | 180,000 | 7,340,000 | 15,100 | 655,000 |
| **Fossil depletion [kg oil eq.]** | 621,000,000 | 2,780,000,000 | 6,880,000,000 | 619,000,000 | 9,040,000 | 85,100,000 | 19,400,000 | 39,000,000 | 1,600,000,000 | 3,660,000 | 154,000,000 |
| **Freshwater consumption [m^3^]** | 8,540,000 | 38,100,000 | 94,600,000 | 8,600,000 | 119,000 | 2,150,000 | 512,000 | 785,000 | 28,900,000 | 59,100 | 2,150,000 |
| **Freshwater ecotoxicity [kg 1,4 DB eq.]** | 42,700,000 | 192,000,000 | 468,000,000 | 41,300,000 | 714,000 | 6,600,000 | 1,420,000 | 3,180,000 | 119,000,000 | 218,000 | 10,500,000 |
| **Freshwater eutrophication [kg P eq.]** | 667,000 | 2,980,000 | 7,420,000 | 672,000 | 9,270 | 103,000 | 21,000 | 47,700 | 1,940,000 | 3,490 | 167,000 |
| **Human toxicity, cancer [kg 1,4-DB eq.]** | 73,000,000 | 328,000,000 | 802,000,000 | 71,700,000 | 1,140,000 | 11,800,000 | 2,600,000 | 6,420,000 | 238,000,000 | 400,000 | 18,200,000 |
| **Human toxicity, non-cancer [kg 1,4-DB eq.]** | 1,500,000,000 | 6,710,000,000 | 16,600,000,000 | 1,490,000,000 | 22,100,000 | 201,000,000 | 44,800,000 | 101,000,000 | 4,030,000,000 | 7,830,000 | 373,000,000 |
| **Ionising radiation [kBq Co-60 eq. to air]** | 249,000,000 | 1,110,000,000 | 2,780,000,000 | 252,000,000 | 3,420,000 | 31,700,000 | 6,240,000 | 16,700,000 | 694,000,000 | 1,310,000 | 62,300,000 |
| **Land use [annual crop eq.·y]** | 28,700,000 | 129,000,000 | 312,000,000 | 28,700,000 | 406,000 | 15,000,000 | 3,090,000 | 2,330,000 | 88,100,000 | 168,000 | 7,370,000 |
| **Marine ecotoxicity [kg 1,4-DB eq.]** | 53,400,000 | 240,000,000 | 585,000,000 | 51,600,000 | 896,000 | 8,280,000 | 1,820,000 | 4,010,000 | 150,000,000 | 273,000 | 13,200,000 |
| **Marine eutrophication [kg N eq.]** | 95,500 | 426,000 | 1,060,000 | 96,300 | 1,330 | 39,900 | 8,370 | 14,700 | 520,000 | 650 | 23,900 |
| **Metal depletion [kg Cu eq.]** | 2,520,000 | 11,400,000 | 27,300,000 | 2,400,000 | 44,500 | 512,000 | 118,000 | 226,000 | 8,070,000 | 14,800 | 623,000 |
| **Photochemical ozone formation, ecosystems [kg NOx eq.]** | 2,200,000 | 9,930,000 | 24,300,000 | 2,130,000 | 33,600 | 368,000 | 91,000 | 149,000 | 6,210,000 | 11,900 | 524,000 |
| **Photochemical ozone formation, human health [kg NOx eq.]** | 2,120,000 | 9,580,000 | 23,400,000 | 2,050,000 | 32,300 | 357,000 | 88,000 | 144,000 | 6,000,000 | 11,500 | 505,000 |
| **Stratospheric ozone depletion [g CFC-11 eq.]** | 1,020,000 | 4,550,000 | 11,300,000 | 1,010,000 | 15,100 | 216,000 | 45,300 | 57,000 | 2,220,000 | 5,670 | 251,000 |
| **Terrestrial acidification [kg SO_2_ eq.]** | 3,180,000 | 14,300,000 | 35,100,000 | 3,130,000 | 47,500 | 637,000 | 143,000 | 224,000 | 8,970,000 | 21,600 | 783,000 |
| **Terrestrial ecotoxicity [kg 1,4-DB eq.]** | 2,540,000,000 | 11,500,000,000 | 27,500,000,000 | 2,410,000,000 | 45,300,000 | 626,000,000 | 193,000,000 | 220,000,000 | 7,740,000,000 | 13,500,000 | 624,000,000 |


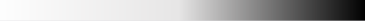


lowest impact highest impact

*As reported by ReCiPe 2016 v1.1 Midpoint (H).

APD, automated peritoneal dialysis; CAPD, continuous ambulatory peritoneal dialysis; CFC, chlorofluorocarbon; CKD, chronic kidney disease; Co-60 eq., cobalt-60 equivalent; tonnes CO_2_e, metric tonnes carbon dioxide equivalents; Cu eq., copper equivalent; DB, 1,4 dichlorobenzene; excl, excluding; HD, haemodialysis; incl, including; m^3^, cubic metre; NOx eq., nitrous oxide equivalent; P eq., phosphate equivalent; PM_2.5_, particulate matter, ≤ 2.5 µm; SC, supportive care; SO_2_, sulphur dioxide; US, United States of America.

**Supplementary Table 12. Environmental impact* was greatest at CKD stage 3 in the UK prevalent population**

|  | **CKD stage** | | | | | | | | | | |
| --- | --- | --- | --- | --- | --- | --- | --- | --- | --- | --- | --- |
|  | **1** | **2** | **3** | **4** | **5: SC** | **5: CAPD** | **5: APD** | **5: at-home HD** | **5: in-centre HD** | **5: peri-transplant** | **5: prevalent transplant** |
| **Climate change, excl biogenic carbon [tonnes CO_2_e]** | 39,700 | 204,000 | 1,190,000 | 145,000 | 1,390 | 5,820 | 6,500 | 6,830 | 124,000 | 1,480 | 52,000 |
| **Climate change, incl biogenic carbon [tonnes CO_2_e]** | 41,200 | 211,000 | 1,240,000 | 151,000 | 1,450 | 4,880 | 5,700 | 7,330 | 131,000 | 1,520 | 54,200 |
| **Fine particulate matter formation [kg PM_2.5_ eq.]** | 28,800 | 145,000 | 828,000 | 98,800 | 928 | 6,610 | 7,710 | 6,270 | 107,000 | 1,180 | 34,200 |
| **Fossil depletion [kg oil eq.]** | 17,300,000 | 89,400,000 | 532,000,000 | 65,000,000 | 627,000 | 2,450,000 | 2,600,000 | 3,220,000 | 56,400,000 | 613,000 | 23,700,000 |
| **Freshwater consumption [m^3^]** | 173,000 | 896,000 | 5,400,000 | 659,000 | 6,350 | 75,700 | 101,000 | 62,700 | 922,000 | 9,630 | 243,000 |
| **Freshwater ecotoxicity [kg 1,4 DB eq.]** | 1,440,000 | 7,220,000 | 40,400,000 | 4,910,000 | 46,000 | 200,000 | 200,000 | 279,000 | 4,670,000 | 52,700 | 1,660,000 |
| **Freshwater eutrophication [kg P eq.]** | 4,210 | 21,400 | 124,000 | 15,000 | 142 | 1,600 | 1,630 | 1,350 | 20,800 | 153 | 5,290 |
| **Human toxicity, cancer [kg 1,4-DB eq.]** | 1,690,000 | 8,450,000 | 47,000,000 | 5,650,000 | 52,500 | 287,000 | 313,000 | 426,000 | 6,800,000 | 68,300 | 1,890,000 |
| **Human toxicity, non-cancer [kg 1,4-DB eq.]** | 33,900,000 | 174,000,000 | 1,020,000,000 | 125,000,000 | 1,210,000 | 4,740,000 | 5,130,000 | 6,310,000 | 108,000,000 | 1,160,000 | 45,200,000 |
| **Ionising radiation [kBq Co-60 eq. to air]** | 4,000,000 | 21,000,000 | 129,000,000 | 15,800,000 | 154,000 | 718,000 | 622,000 | 1,680,000 | 25,600,000 | 136,000 | 5,930,000 |
| **Land use [annual crop eq.·y]** | 947,000 | 4,850,000 | 28,800,000 | 3,470,000 | 32,700 | 594,000 | 525,000 | 332,000 | 5,140,000 | 40,000 | 1,240,000 |
| **Marine ecotoxicity [kg 1,4-DB eq.]** | 1,840,000 | 9,250,000 | 51,700,000 | 6,280,000 | 59,000 | 256,000 | 262,000 | 360,000 | 6,010,000 | 67,400 | 2,130,000 |
| **Marine eutrophication [kg N eq.]** | 2,180 | 11,500 | 69,900 | 8,630 | 85 | 1,440 | 1,270 | 1,340 | 19,800 | 110 | 3,250 |
| **Metal depletion [kg Cu eq.]** | 98,900 | 490,000 | 2,680,000 | 323,000 | 2,980 | 17,300 | 18,500 | 21,400 | 352,000 | 4,180 | 106,000 |
| **Photochemical ozone formation, ecosystems [kg NOx eq.]** | 63,800 | 323,000 | 1,850,000 | 218,000 | 2,050 | 10,700 | 12,500 | 12,500 | 236,000 | 2,480 | 75,600 |
| **Photochemical ozone formation, human health [kg NOx eq.]** | 61,200 | 310,000 | 1,770,000 | 209,000 | 1,960 | 10,400 | 12,100 | 12,100 | 228,000 | 2,380 | 72,500 |
| **Stratospheric ozone depletion [g CFC-11 eq.]** | 32,900 | 171,000 | 1,020,000 | 125,000 | 1,210 | 7,660 | 7,050 | 4,820 | 80,800 | 1,130 | 45,900 |
| **Terrestrial acidification [kg SO_2_ eq.]** | 70,500 | 357,000 | 2,040,000 | 244,000 | 2,300 | 17,800 | 18,800 | 15,100 | 260,000 | 3,010 | 84,800 |
| **Terrestrial ecotoxicity [kg 1,4-DB eq.]** | 99,200,000 | 490,000,000 | 2,660,000,000 | 319,000,000 | 2,930,000 | 21,900,000 | 31,200,000 | 21,500,000 | 348,000,000 | 3,910,000 | 103,000,000 |


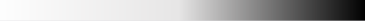


lowest impact highest impact

*As reported by ReCiPe 2016 v1.1 Midpoint (H).

APD, automated peritoneal dialysis; CAPD, continuous ambulatory peritoneal dialysis; CFC, chlorofluorocarbon; CKD, chronic kidney disease; Co-60 eq., cobalt-60 equivalent; tonnes CO_2_e, metric tonnes carbon dioxide equivalents; Cu eq., copper equivalent; DB, 1,4 dichlorobenzene; excl, excluding; HD, haemodialysis; incl, including; m^3^, cubic metre; NOx eq., nitrous oxide equivalent; P eq., phosphate equivalent; PM_2.5_, particulate matter, ≤ 2.5 µm; SC, supportive care; SO_2_, sulphur dioxide; UK, United Kingdom.

**Supplementary Table 13. Exploratory analyses: GHG emissions per population**

| **Country** | Dataset  used | **CKD stage (tonnes CO_2_e)** | | | | | | | | | | | |
| --- | --- | --- | --- | --- | --- | --- | --- | --- | --- | --- | --- | --- | --- |
|  |  | **1** | **2** | **3** | **4** | **5:SC** | **5: PD (CAPD)** | **5: PD (APD)** | **5: at-home HD** | **5: in-centre HD** | **5: peri-transplant** | **5: prevalent transplant** | **Total GHG emissions (tonnes CO_2_e)** |
| **Australia** | UK | 27,100 | 111,000 | 339,000 | 49,100 | 1,100 | 4,610 | 13,900 | 12,200 | 118,000 | 1,750 | 29,500 | **708,000** |
|  | USA | 66,800 | 260,000 | 693,000 | 92,900 | 911 | 4,570 | 13,300 | 12,600 | 123,000 | 1,830 | 22,700 | **1,290,000** |
| **Belgium** | UK | 5,730 | 30,600 | 176,000 | 42,900 | 243 | 592 | 1,200 | 258 | 32,600 | 411 | 6,960 | **298,000** |
|  | USA | 14,700 | 76,700 | 424,000 | 108,000 | 216 | 614 | 1,150 | 278 | 35,300 | 344 | 5,290 | **666,000** |
| **Brazil** | UK | 84,500 | 595,000 | 3,580,000 | 349,000 | 4,630 | 5,710 | 38,100 | 0 | 949,000 | 2,560 | 70,000 | **5,680,000** |
|  | USA | 192,000 | 1,370,000 | 8,130,000 | 815,000 | 4,520 | 6,180 | 37,700 | 0 | 1,020,000 | 1,670 | 50,900 | **11,600,000** |
| **Germany** | UK | 141,000 | 186,000 | 526,000 | 55,500 | 1,740 | 5,050 | 9,550 | 2,870 | 398,000 | 413 | 29,600 | **1,360,000** |
|  | USA | 345,000 | 455,000 | 1,250,000 | 137,000 | 1,650 | 5,400 | 9,370 | 3,090 | 425,000 | 297 | 22,300 | **2,650,000** |
| **Italy** | UK | 20,900 | 84,000 | 356,000 | 64,500 | 1,300 | 3,380 | 6,270 | 1,710 | 222,000 | 4,500 | 30,800 | **795,000** |
|  | USA | 49,900 | 198,000 | 815,000 | 154,000 | 1,170 | 3,500 | 5,990 | 1,820 | 236,000 | 3,530 | 22,800 | **1,490,000** |
| **Japan** | UK | 91,500 | 1,710,000 | 5,010,000 | 776,000 | 12,300 | 15,400 | 23,800 | 5,640 | 2,930,000 | 721 | 27,400 | **10,600,000** |
|  | USA | 241,000 | 4,350,000 | 12,100,000 | 1,950,000 | 10,400 | 16,000 | 23,300 | 6,120 | 3,160,000 | 646 | 20,000 | **21,900,000** |
| **Netherlands** | UK | 11,000 | 63,600 | 258,000 | 24,500 | 393 | 1,150 | 2,750 | 1,800 | 30,300 | 198 | 16,900 | **410,000** |
|  | USA | 29,200 | 164,000 | 632,000 | 62,700 | 343 | 1,200 | 2,670 | 1,930 | 32,600 | 172 | 12,800 | **940,000** |
| **Spain** | UK | 18,300 | 130,000 | 912,000 | 80,300 | 1,380 | 11,100 | 5,470 | 299 | 146,000 | 1,130 | 45,500 | **1,350,000** |
|  | USA | 48,200 | 336,000 | 2,260,000 | 205,000 | 1,290 | 11,900 | 5,400 | 330 | 159,000 | 881 | 34,200 | **3,060,000** |

APD, automated peritoneal dialysis; CAPD, continuous ambulatory peritoneal dialysis; CKD, chronic kidney disease; CO_2_e, carbon dioxide equivalents; GHG, greenhouse gas; HD, haemodialysis; SC, supportive care; UK, United Kingdom; USA, United States of America.

**Supplementary Table 14. Sensitivity analysis showed how altering the different model parameters in the USA affected annual GHG emissions per population at each CKD stage**

| **Model parameter** | **GHG emissions (tonnes CO_2_e) (% difference vs base case)** | | | | | | | | | | |
| --- | --- | --- | --- | --- | --- | --- | --- | --- | --- | --- | --- |
|  | **1** | **2** | **3** | **4** | **5: SC** | **5: CAPD** | **5: APD** | **5: at-home HD** | **5: in-centre HD** | **5: peri-transplant** | **5: prevalent transplant** |
| ***Base case*** | 1,480,000 | 6,620,000 | 16,300,000 | 1,460,000 | 21,900 | 211,000 | 47,500 | 95,100 | 3,900,000 | 9,080 | 366,000 |
| **50% telemedicine** | 1,380,000 (93.3%) | 6,160,000 (93.1%) | 15,200,000 (93.4%) | 1,370,000 (94.0%) | 19,700 (89.9%) | 203,000 (95.8%) | 46,100 (97.0%) | 93,100 (97.9%) | 3,810,000 (97.9%) | 8,680 (95.6%) | 348,000 (95.1%) |
| **50% electric vehicles** | 1,430,000 (97.1%) | 6,430,000 (97.1%) | 15,900,000 (97.5%) | 1,420,000 (97.5%) | 21,000 (95.6%) | 207,000 (98.1%) | 46,900 (98.8%) | 94,200 (99.1%) | 3,760,000 (96.6%) | 8,900 (97.9%) | 355,000 (97.1%) |
| **50% heating/cooling/lighting** | 984,000 (66.7%) | 4,410,000 (66.6%) | 10,800,000 (66.1%) | 961,000 (65.7%) | 15,200 (69.4%) | 158,000 (74.9%) | 37,500 (79.0%) | 80,500 (84.6%) | 3,060,000 (78.5%) | 6,560 (72.2%) | 242,000 (66.3%) |
| **150% heating/cooling/lighting** | 1,970,000 (133.3%) | 8,820,000 (133.3%) | 21,900,000 (134.0%) | 1,960,000 (134.1%) | 28,700 (130.8%) | 265,000 (125.2%) | 57,500 (121.0%) | 110,000 (115.5%) | 4,730,000 (121.5%) | 11,600 (127.9%) | 490,000 (134.0%) |
| **50% travel distance** | 1,370,000 (92.8%) | 6,120,000 (92.6%) | 15,200,000 (92.9%) | 1,370,000 (93.7%) | 19,700 (89.9%) | 202,000 (95.5%) | 46,000 (96.8%) | 92,900 (97.6%) | 3,520,000 (90.4%) | 7,800 (85.9%) | 346,000 (94.5%) |
| **150% travel distance** | 1,580,000 (107.2%) | 7,100,000 (107.3%) | 17,500,000 (107.2%) | 1,560,000 (106.5%) | 24,200 (110.4%) | 221,000 (104.6%) | 49,100 (103.2%) | 97,300 (102.3%) | 4,270,000 (109.6%) | 10,400 (114.0%) | 387,000 (105.8%) |
| **50% hospital**  **stay** | 874,000 (59.2%) | 3,940,000 (59.5%) | 9,570,000 (58.6%) | 846,000 (57.9%) | 13,900 (63.1%) | 162,000 (76.7%) | 39,900 (84.0%) | 81,200 (85.4%) | 3,340,000 (85.6%) | 6,690 (73.6%) | 213,000 (58.1%) |
| **150% hospital**  **stay** | 2,080,000 (140.8%) | 9,320,000 (140.9%) | 23,100,000 (141.5%) | 2,080,000 (142.2%) | 30,100 (137.1%) | 261,000 (123.4%) | 55,200 (116.1%) | 109,000 (114.7%) | 4,460,000 (114.4%) | 11,500 (126.3%) | 520,000 (142.0%) |
| **50% health visits** | 866,000 (58.7%) | 3,900,000 (59.0%) | 9,500,000 (58.2%) | 838,000 (57.3%) | 13,700 (62.6%) | 162,000 (76.5%) | 39,800 (83.8%) | 81,000 (85.2%) | 3,330,000 (85.5%) | 6,670 (73.4%) | 211,000 (57.5%) |
| **150% health visits** | 2,090,000 (141.3%) | 9,320,000 (140.9%) | 23,200,000 (141.9%) | 2,080,000 (142.5%) | 30,200 (137.7%) | 261,000 (123.6%) | 55,200 (116.2%) | 109,000 (114.8%) | 4,910,000 (126.0%) | 11,500 (126.5%) | 522,000 (142.7%) |
| **Including medicine** | 1,720,000 (116.8%) | 7,670,000 (116.0%) | 18,800,000 (115.1%) | 1,650,000 (112.6%) | 29,000 (132.0%) | 259,000 (122.6%) | 54,900 (115.5%) | 103,000 (108.7%) | 4,230,000 (108.5%) | 10,400 (114.5%) | 517,000 (141.3%) |

APD, automated peritoneal dialysis; CAPD, continuous ambulatory peritoneal dialysis; CKD, chronic kidney disease; CO_2_e, carbon dioxide equivalents; HD, haemodialysis; SC, supportive care; USA, United States of America.

**Supplementary Table 15. Sensitivity analysis showed how altering the different model parameters in the UK affected annual GHG emissions per population at each CKD stage**

| **Model parameter** | **GHG emissions (tonnes CO_2_e) (% difference vs base case)** | | | | | | | | | | |
| --- | --- | --- | --- | --- | --- | --- | --- | --- | --- | --- | --- |
|  | **1** | **2** | **3** | **4** | **5: SC** | **5: CAPD** | **5: APD** | **5: at-home HD** | **5: in-centre HD** | **5: peri-transplant** | **5: prevalent transplant** |
| ***Base case*** | 39,700 | 204,000 | 1,190,000 | 145,000 | 1,390 | 5,820 | 6,500 | 6,830 | 124,000 | 1,480 | 52,000 |
| **50% telemedicine** | 35,800 (90.2%) | 185,000 (90.9%) | 1,100,000 (92.3%) | 134,000 (92.4%) | 1,290 (92.8%) | 5,670 (97.3%) | 6,390 (98.3%) | 6,740 (98.7%) | 122,000 (98.6%) | 1,260 (85.1%) | 49,000 (94.3%) |
| **50% Electric vehicles** | 37,600 (94.5%) | 194,000 (95.1%) | 1,140,000 (95.7%) | 139,000 (95.8%) | 1,340 (96.7%) | 5,740 (98.5%) | 6,450 (99.2%) | 6,780 (99.3%) | 117,000 (94.5%) | 1,390 (94.2%) | 50,300 (96.7%) |
| **50% heating/cooling/lighting** | 30,400 (76.4%) | 154,000 (75.7%) | 885,000 (74.2%) | 107,000 (73.8%) | 1,010 (73.0%) | 4,790 (82.2%) | 5,670 (87.2%) | 6,170 (90.4%) | 106,000 (85.7%) | 1,190 (80.5%) | 37,500 (72.1%) |
| **150% heating/cooling/lighting** | 49,100 (123.6%) | 254,000 (124.5%) | 1,500,000 (125.8%) | 183,000 (126.4%) | 1,770 (127.4%) | 6,860 (117.8%) | 7,330 (112.8%) | 7,480 (109.6%) | 141,000 (114.2%) | 1,760 (119.3%) | 66,600 (128.1%) |
| **50% travel distance** | 35,700 (89.7%) | 184,000 (90.5%) | 1,100,000 (91.8%) | 133,000 (91.9%) | 1,280 (92.0%) | 5,640 (96.9%) | 6,390 (98.3%) | 6,730 (98.5%) | 105,000 (84.6%) | 1,180 (80.1%) | 48,600 (93.5%) |
| **150% travel distance** | 43,800 (110.3%) | 223,000 (109.4%) | 1,290,000 (108.4%) | 157,000 (108.1%) | 1,500 (107.7%) | 6,010 (103.1%) | 6,610 (101.7%) | 6,930 (101.5%) | 143,000 (115.4%) | 1,770 (119.6%) | 55,300 (106.3%) |
| **50% Hospital**  **stay** | 26,100 (65.8%) | 132,000 (64.6%) | 744,000 (62.3%) | 89,600 (61.8%) | 841 (60.6%) | 4,650 (79.9%) | 5,730 (88.2%) | 6,120 (89.6%) | 111,000 (90.0%) | 1,170 (79.4%) | 30,700 (59.1%) |
| **150% Hospital**  **stay** | 53,400 (134.3%) | 276,000 (135.3%) | 1,640,000 (137.7%) | 201,000 (138.3%) | 1,930 (139.2%) | 6,980 (119.8%) | 7,270 (111.9%) | 7,540 (110.4%) | 136,000 (110.1%) | 1,780 (120.4%) | 73,300 (141.0%) |
| **50% health visits** | 26,000 (65.3%) | 131,000 (64.2%) | 738,000 (61.8%) | 88,800 (61.2%) | 832 (59.9%) | 4,650 (79.9%) | 5,710 (87.9%) | 6,110 (89.4%) | 111,000 (89.8%) | 1,170 (79.0%) | 30,400 (58.4%) |
| **150% health visits** | 53,500 (134.7%) | 277,000 (135.9%) | 1,650,000 (138.1%) | 201,000 (138.3%) | 1,940 (140.0%) | 7,000 (120.2%) | 7,280 (112.1%) | 7,550 (110.6%) | 137,000 (110.3%) | 1,780 (120.8%) | 73,700 (141.8%) |
| **Including medicine** | 68,300 (171.8%) | 339,000 (166.1%) | 1,900,000 (159.5%) | 220,000 (151.4%) | 2,000 (143.9%) | 7,630 (131.0%) | 7,690 (118.3%) | 7,750 (113.4%) | 140,000 (113.0%) | 1,820 (123.4%) | 77,500 (149.0%) |

APD, automated peritoneal dialysis; CAPD, continuous ambulatory peritoneal dialysis; CKD, chronic kidney disease; CO_2_e, carbon dioxide equivalents; HD, haemodialysis; SC, supportive care; UK, United Kingdom.

**Supplementary Table 16. Environmental impact of CKD: a summary of the key findings, implications, and recommended actions**

| **Key finding** | **Implication** | **Recommended actions** |
| --- | --- | --- |
| GHG emissions increase with CKD stage, peaking with in-centre HD | Progression of CKD significantly increases environmental burden | Prioritise early detection and intervention to slow CKD progression |
| Hospitalisations are a major driver of emissions across all stages | Preventable events contribute significantly to environmental impact | Improve management of comorbidities (e.g., heart failure, infections, AKI); use preventive strategies. Prescription of GDMT |
| Transport emissions are substantial due to frequent healthcare visits | Patient travel significantly adds to GHG emissions | Promote telehealth, active/public transport, carpooling; coordinate multidisciplinary clinic days |
| In-centre HD has highest emissions; PD and transplant are lower-emission alternatives | Treatment modality heavily influences GHG emissions | Prioritise transplantation and PD where appropriate; support ‘PD-first’ approaches |
| CAPD emits less GHG emissions than APD | Treatment choice within modality affects GHG emissions | Encourage CAPD over APD when clinically appropriate |
| Transplant and supportive kidney care underutilised globally | Limited access to lower-impact options | Expand access through policy, legislation, education, and global guidelines |
| Dialysis units slow to adopt sustainable practices | Lack of environmental standards and accountability | Implement environmental regulations; foster leadership (e.g., Sustainability Champions); incentivise innovation |
| CKD-related emissions include harmful pollutants (e.g., PM_2.5_), not just GHGs | Broader health and environmental consequences | Expand scope of environmental evaluations beyond GHGs; integrate findings into health policy |
| International variation in emissions due to systemic and geographic factors | Need for context-specific strategies | Develop national data infrastructure; support country-level environmental policy planning |
| Lack of environmental data for medications and healthcare usage | Environmental impact may be underestimated | Improve data transparency and inclusion of pharmaceuticals in environmental impact assessments |
| Environmental burden focused only on diagnosed CKD patients | Total emissions likely underestimated | Strengthen screening efforts and improve CKD detection and diagnosis |

AKI, acute kidney injury; APD, automated peritoneal dialysis; CAPD, continuous ambulatory peritoneal dialysis; CKD, chronic kidney disease; GDMT, guideline-directed medical therapies; GHG, greenhouse gas emission; HD, haemodialysis; PD, peritoneal dialysis.

**Supplementary References**

S1. Flaxman P. The 10-minute appointment. *Br J Gen Pract*. 2015;65(640):573-574. doi:10.3399/bjgp15X687313

S2. McGain F, Burnham JP, Lau R, Aye L, Kollef MH, McAlister S. The carbon footprint of treating patients with septic shock in the intensive care unit. *Critical Care and Resuscitation*. 2018;20(4):304-312.

S3. Arulkumaran N, Annear NMP, Singer M. Patients with end-stage renal disease admitted to the intensive care unit: systematic review. *British Journal of Anaesthesia*. 2013;110(1):13-20. doi:10.1093/BJA/AES401

S4. Weiss DJ, Nelson A, Vargas-Ruiz CA, et al. Global maps of travel time to healthcare facilities. *Nature Medicine*. 2020;26(12):1835-1838. doi:10.1038/s41591-020-1059-1

S5. Eyjolfsson R. Design and Manufacture of Pharmaceutical Tablets. *Design and Manufacture of Pharmaceutical Tablets*. Published online October 9, 2014:1-55. doi:10.1016/C2014-0-02382-9

S6. Reker D, Blum SM, Steiger C, et al. Inactive ingredients in oral medications. *Science Translational Medicine*. 2019;11(483). doi:10.1126/scitranslmed.aau6753

S7. Sharma RK, Sarkar P, Singh H. Assessing the sustainability of a manufacturing process using life cycle assessment technique—a case of an Indian pharmaceutical company. *Clean Technologies and Environmental Policy*. 2020;22(6):1269-1284. doi:10.1007/S10098-020-01865-4

S8. Hadinoto K, Tran TT, Chua A, Cheow WS. Comparing environmental impacts of direct compaction versus wet granulation tableting methods for drugs with poor flowability by life cycle assessment. *Chemical Engineering Research and Design*. 2022;183:439-451. doi:10.1016/J.CHERD.2022.05.029

S9. Raju G, Sarkar P, Singla E, Singh H, Sharma RK. Comparison of environmental sustainability of pharmaceutical packaging. *Perspectives in Science*. 2016;8:683-685. doi:10.1016/J.PISC.2016.06.058

S10. Frapak. 50ml PET bottles F523A - 28 ROPP PET bottle amber. 2022. Accessed July 27, 2022. https://www.frapak.com/en/bottles/50ml-pet-bottle-standard-round-28-ropp-in-stock-amber/F523AEP04/

S11. Benavides PT, Dunn JB, Han J, Biddy M, Markham J. Exploring Comparative Energy and Environmental Benefits of Virgin, Recycled, and Bio-Derived PET Bottles. *ACS Sustainable Chemistry and Engineering*. 2018;6(8):9725-9733. doi:10.1021/ACSSUSCHEMENG.8B00750/ASSET/IMAGES/LARGE/SC-2018-007507_0005.JPEG

S12. Bruijn, H, Duin, R, Huijbregts, MAJ, et al. *Handbook on Life Cycle Assessment: Operational Guide to the ISO Standards*.; 2002. Accessed August 7, 2025. https://link.springer.com/book/10.1007/0-306-48055-7

S13. Huijbregts MAJ, Steinmann ZJN, Elshout PMF, et al. ReCiPe2016: a harmonised life cycle impact assessment method at midpoint and endpoint level. *Int J Life Cycle Assess*. 2017;22(2):138-147. doi:10.1007/s11367-016-1246-y

S14. Australia and New Zealand Dialysis and Transplant Registry. ANZDATA 44th Annual Report 2021. 2021. Accessed August 23, 2022. https://www.anzdata.org.au/report/anzdata-44th-annual-report-2021-data-to-2020/

S15. Nerbass FB, Saldanha Thomé F, Merege O, Neto V, Lugon JR, Sesso R. Brazilian Dialysis Survey 2020 Censo Brasileiro de Diálise 2020 Helbert do Nascimento Lima 2. *Braz J Nephrol (J Bras Nefrol)*. Published online 2022. doi:10.1590/2175-8239

S16. Nitta K, Goto S, Masakane I, et al. Annual dialysis data report for 2018, JSDT Renal Data Registry: Survey methods, facility data, incidence, prevalence, and mortality. *Renal Replacement Therapy*. 2020;6(1):1-18. doi:10.1186/S41100-020-00286-9/FIGURES/18

S17. ERA-EDTA. ERA_EDTA Registry Annual Report 2019. 2021. https://www.era-online.org/wp-content/uploads/2022/11/ERA-Registry-Annual-Report-2019.pdf

S18. United States Renal Data System. USRDS Annual Data Report: Epidemiology of kidney disease in the United States. 2021. https://adr.usrds.org/2021

S19. Welch JL, Meek J, Bartlett Ellis RJ, Ambuehl R, Decker BS. PATTERNS OF HEALTHCARE ENCOUNTERS EXPERIENCED BY PATIENTS WITH CHRONIC KIDNEY DISEASE. *Journal of Renal Care*. 2017;43(4):209-218. doi:10.1111/jorc.12200

S20. Thorsteinsdottir B, Ramar P, Hickson LJ, et al. Care of the dialysis patient: Primary provider involvement and resource utilization patterns - a cohort study. *BMC Nephrology*. 2017;18(1). doi:10.1186/S12882-017-0728-X

S21. Lovasik BP, Zhang R, Hockenberry JM, et al. Emergency department use among kidney transplant recipients in the United States. *American Journal of Transplantation*. 2018;18(4):868-880. doi:10.1111/AJT.14578

S22. Strijack B, Mojica J, Sood M, et al. Outcomes of Chronic Dialysis Patients Admitted to the Intensive Care Unit. *Journal of the American Society of Nephrology*. 2009;20(11):2441-2447. doi:10.1681/ASN.2009040366

S23. Abrol N, Kashyap R, Frank RD, et al. Preoperative Factors Predicting Admission to the Intensive Care Unit After Kidney Transplantation. *Mayo Clinic Proceedings: Innovations, Quality & Outcomes*. 2019;3(3):285. doi:10.1016/J.MAYOCPIQO.2019.06.008

S24. Baker RJ, Stevens KK, Palmer N. Post-Operative Care in the Kidney Transplant Recipient: Clinical Practice Guidelines.

S25. Hutchison CA, Crowe AV, Stevens PE, Harrison DA, Lipkin GW. Case mix, outcome and activity for patients admitted to intensive care units requiring chronic renal dialysis: a secondary analysis of the ICNARC Case Mix Programme Database. *Critical care (London, England)*. 2007;11(2). doi:10.1186/CC5785

S26. Okidi OO, Van Dellen D, Sobajo C, Summers A, Greer JR, Augustine T. Kidney transplant recipients requiring critical care admission within one year of transplant. *Experimental and Clinical Transplantation*. 2017;15(1):40-46. doi:10.6002/ECT.2015.0356
